# Young osteocyte-derived extracellular vesicles facilitate osteogenesis by transferring tropomyosin-1

**DOI:** 10.1186/s12951-024-02367-x

**Published:** 2024-04-25

**Authors:** Zhen-Xing Wang, Xiao Lin, Jia Cao, Yi-Wei Liu, Zhong-Wei Luo, Shan-Shan Rao, Qiang Wang, Yi-Yi Wang, Chun-Yuan Chen, Guo-Qiang Zhu, Fu-Xing-Zi Li, Yi-Juan Tan, Yin Hu, Hao Yin, You-You Li, Ze-Hui He, Zheng-Zhao Liu, Ling-Qing Yuan, Yong Zhou, Zheng-Guang Wang, Hui Xie

**Affiliations:** 1 Department of Orthopedics, Movement System Injury and Repair Research Center, Xiangya Hospital, Central South University, Changsha, 410008 Hunan China; 2 Hunan Key Laboratory of Angmedicine, Changsha, 410008 Hunan China; 3 National Clinical Research Center for Geriatric Disorders (Xiangya Hospital), Changsha, 410008 Hunan China; 4 The Second Xiangya Hospital, Central South University, Changsha, 410008 Hunan China; 5 Department of Laboratory Medicine, Affiliated Zhejiang Hospital, Zhejiang University School of Medicine, Hangzhou, 310013 Zhejiang China; 6 Third Xiangya Hospital, Central South University, Changsha, 410013 Hunan China

**Keywords:** Osteocytes, Extracellular vesicles, Bone marrow messenchymal stem cells, Osteogenesis, Tropomyosin-1

## Abstract

**Background:**

Bone marrow mesenchymal stem cells (BMSCs) can undergo inadequate osteogenesis or excessive adipogenesis as they age due to changes in the bone microenvironment, ultimately resulting in decreased bone density and elevated risk of fractures in senile osteoporosis. This study aims to investigate the effects of osteocyte senescence on the bone microenvironment and its influence on BMSCs during aging.

**Results:**

Primary osteocytes were isolated from 2-month-old and 16-month-old mice to obtain young osteocyte-derived extracellular vesicles (YO-EVs) and senescent osteocyte-derived EVs (SO-EVs), respectively. YO-EVs were found to significantly increase alkaline phosphatase activity, mineralization deposition, and the expression of osteogenesis-related genes in BMSCs, while SO-EVs promoted BMSC adipogenesis. Neither YO-EVs nor SO-EVs exerted an effect on the osteoclastogenesis of primary macrophages/monocytes. Our constructed transgenic mice, designed to trace osteocyte-derived EV distribution, revealed abundant osteocyte-derived EVs embedded in the bone matrix. Moreover, mature osteoclasts were found to release osteocyte-derived EVs from bone slices, playing a pivotal role in regulating the functions of the surrounding culture medium. Following intravenous injection into young and elderly mouse models, YO-EVs demonstrated a significant enhancement of bone mass and biomechanical strength compared to SO-EVs. Immunostaining of bone sections revealed that YO-EV treatment augmented the number of osteoblasts on the bone surface, while SO-EV treatment promoted adipocyte formation in the bone marrow. Proteomics analysis of YO-EVs and SO-EVs showed that tropomyosin-1 (TPM1) was enriched in YO-EVs, which increased the matrix stiffness of BMSCs, consequently promoting osteogenesis. Specifically, the siRNA-mediated depletion of *Tpm1* eliminated pro-osteogenic activity of YO-EVs both in vitro and in vivo.

**Conclusions:**

Our findings suggested that YO-EVs played a crucial role in maintaining the balance between bone resorption and formation, and their pro-osteogenic activity declining with aging. Therefore, YO-EVs and the delivered TPM1 hold potential as therapeutic targets for senile osteoporosis.

**Graphical abstract:**

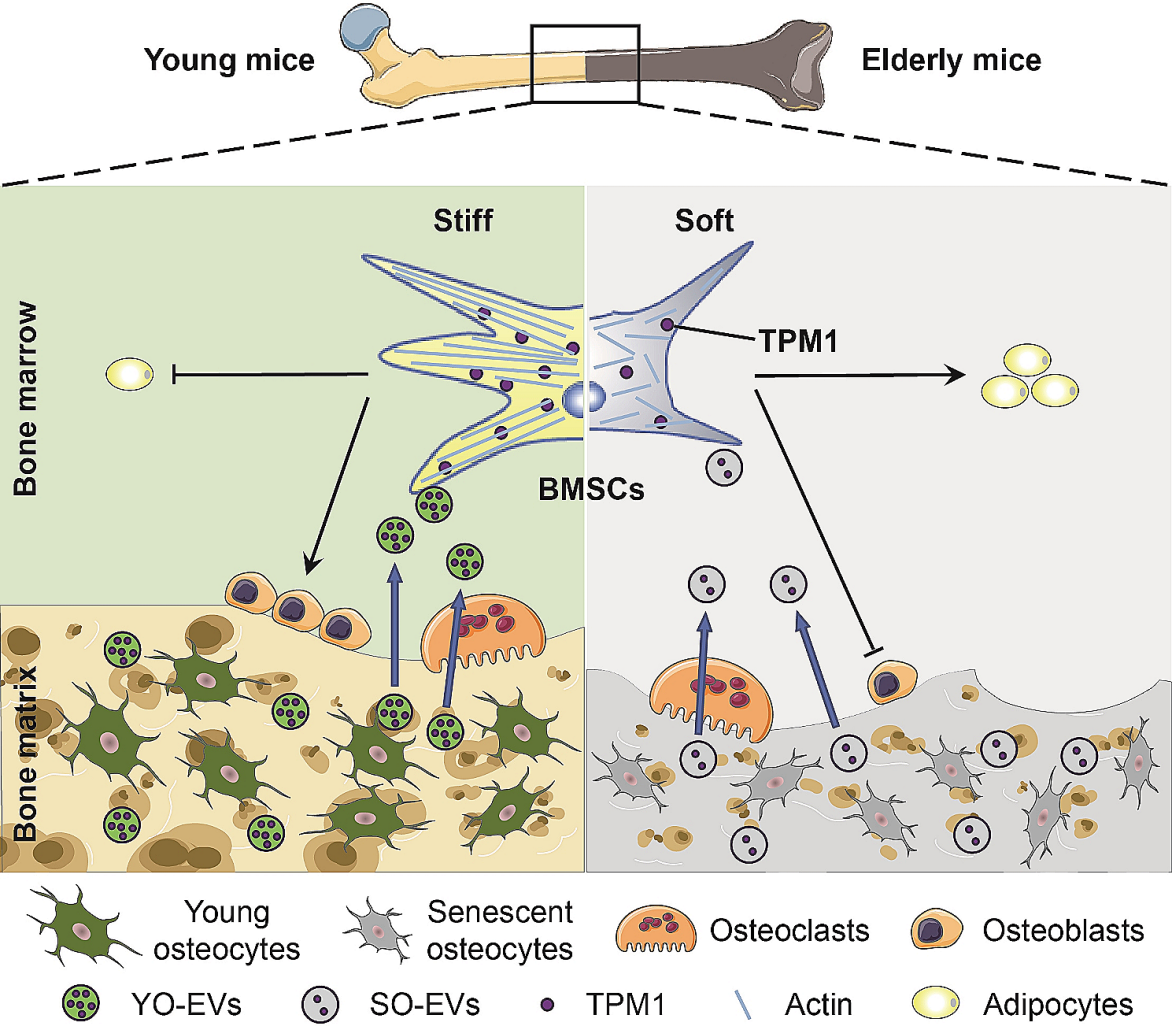

**Supplementary Information:**

The online version contains supplementary material available at 10.1186/s12951-024-02367-x.

## Introduction

Osteoporosis is a prevalent age-related disorder, characterized by the progressive loss of bone mass and quality, leading to an elevated risk of bone fractures and associated morbidity and mortality [1, 2]. Bone marrow mesenchymal stem cells (BMSCs) are capable of differentiating into various cell types, including osteoblasts and adipocytes. However, with individual aging, the capacity of BMSCs to differentiate into osteoblasts decreases, while their differentiation towards adipocytes increases [3–5]. This age-related alteration is closely linked to changes in the intracellular matrix, which is critical to their shape, motility, and differentiation [6]. This alteration, particularly the actin cytoskeleton, plays an essential role in regulating BMSC differentiation. The cytoskeleton of BMSCs is involved in osteogenic differentiation by regulating intracellular signaling, gene expression, and extracellular matrix deposition [7]. In aged BMSCs, there is a marked reduction in F-actin levels that leads to modulation of RhoA and BMP2/Smad signaling, which subsequently leads to decreased osteogenesis and increased adipogenesis [8, 9]. Thus, these age-related changes in the bone microenvironment can contribute to a decline in bone strength and an increased risk of fractures.

Bone is a highly dynamic organ characterized by continuous remodeling through a dynamic interplay between bone formation and resorption, a phenomenon referred to as bone-remodeling coupling [10, 11]. This intricate process is regulated by a diverse array of factors, such as growth factors, cytokines, and hormones, which modulate the balance between bone formation and resorption. The precise coordination of bone formation and bone resorption is essential to maintain bone homeostasis through the bone remodeling process [12]. Importantly, osteoclastic bone resorption prompts the release of multiple growth factors from the bone matrix that are known to promote bone formation [13, 14]. Previous studies have revealed that the active release of transforming growth factor-β1 (TGF-β1) and insulin-like growth factor 1 (IGF-1) from the skeleton matrix occurs during osteoclastic bone resorption [15, 16]. While TGF-β1 is responsible for recruiting BMSCs to bone resorption sites, IGF-1 stimulates their differentiation into osteoblasts. These studies strongly suggest that vital signaling molecules are deposited within the bone matrix.

The bone matrix contains an abundance of osteocytes, making them the predominant cells in bone that are responsible for maintaining bone health through sensing and responding to mechanical and biological signals within the bone microenvironment [17]. Osteocytes are embedded within the bone matrix, forming a vast network of interconnected cells that facilitate communication between bone-forming osteoblasts and bone-resorbing osteoclasts [18]. Osteocytes play a pivotal role in regulating the activity of both osteoblasts and osteoclasts and are thereby essential for bone remodeling [19, 20]. Furthermore, recent research has shown that important factors implicated in bone remodeling are packaged in bilayer membrane vesicles with a spherical shape [21]. Biologically active molecules are transferred between adjacent or distant cells by these organelles.

Extracellular vesicles (EVs) are small (average diameter of 40–200 nm) membrane-derived structures released into the extracellular space by various cell types [22]. These lipid-based carriers have been identified as efficient vehicles for transferring proteins, RNAs, and lipids between cells, delivering their contents to target cells via endocytosis and membrane fusion. Recent research has underscored the crucial significance of bone-derived EVs in the regulation of bone remodeling, primarily by facilitating the transfer of vital molecules that are indispensable for the differentiation and intercellular communication of osteoclasts and osteoblasts [23, 24]. Studies on EVs in bone metabolism and the bone microenvironment have been limited. However, there is mounting evidence suggesting that osteocyte-derived EVs play a crucial function in regulating bone remodeling, angiogenesis, and mineralization by transporting various bioactive molecules [21, 25]. Our previous studies have reported the impact of EVs embedded in the aged bone matrix; specifically, it was observed that these EVs facilitated fat accumulation and stimulated vascular calcification, a phenomenon referred to as the “calcification paradox” [26]. In contrast, young osteocyte-derived EVs showed potential for transfer to brain tissue and maintenance of the health and functionality of metal behaviors [27]. Thus, the role of osteocyte-derived EVs in mediating interactions between bone cells, and the mechanisms underlying such communications, are of great interest. A more thorough understanding of these changes may provide valuable insights into potential therapeutics for age-related bone loss and osteoporosis.

This study aims to investigate the relationship between osteocyte-derived EVs in the bone matrix and the differentiation fate of BMSCs in the bone marrow and to determine how these factors interact to regulate differentiation. In this study, we isolated, purified, and identified young osteocyte-derived EVs (YO-EVs) and senescent osteocyte-derived EVs (SO-EVs) to examine their effects on osteogenic and adipogenic differentiation. In the bone matrix, osteocyte-derived EVs were traced by our constructed transgene mice and released by mature osteoclasts to assess the impact on BMSCs. The study also explored the differential effects of YO-EVs *versus* SO-EVs on promoting osteogenesis in vivo using young and anile mouse models. Additionally, proteomics analysis was conducted to identify key molecules enriched in YO-EVs that promoted bone formation, with tropomyosin-1 (TPM1) being identified as a prominent molecule that increased matrix stiffness and benefited osteogenesis [28] compared to SO-EVs. Specific knockdown of TPM1 significantly decreased the pro-osteogenesis functions of YO-EVs both in vitro and in vivo. In particular, we elucidated the specific mechanisms by which YO-EVs transferred TPM1 to regulate intracellular matrix stiffness and promote osteogenic differentiation. Such a comprehensive understanding of the age-related changes contributing to osteoporosis is essential in devising effective interventions for improving bone health in older adults and reducing the burden of osteoporosis.

## Materials and methods

### Ethics statement

The animal study was conducted in accordance with the guidelines outlined in the Care and Use of Laboratory Animals and received formal approval from the Ethical Review Board at Xiangya Hospital of Central South University. The animals were kept in specific pathogen-free (SPF) conditions, adhering to a 12/12 hour light-dark cycle at a temperature of 22°C and a humidity level of 50 − 55%. They were given unrestricted access to both food pellets and tap water.

### Extraction and culture of primary cells

Primary osteocytes were extracted from the bone matrix of male C57BL/6 mice, specifically from the femurs and tibias, as previously documented [27]. Primary young osteocytes (YO) or senescent osteocytes (SO) were isolated from 2-month-old or 16-month-old mice, respectively. The age of the mice was used as the primary criterion for categorizing osteocytes as “young” or “senescent”. The femora and tibiae were dissected under aseptic conditions, with the periosteum, bone epiphyses, and bone marrow meticulously eliminated using minimum essential medium with alpha modification (α-MEM; Cat. No. PM150421, Procell) supplemented with 2% penicillin and streptomycin (P/S; Cat. No. 15,070,063, Gibco). Subsequently, the bone was crushed into small pieces, approximately 1 − 2 mm in length. These bone fragments were then incubated three times for 30 min at 37 °C in α-MEM containing 10 units mL^− 1^ collagenase II (Cat. No. 17,101,015, Gibco). Next, the bone pieces were subjected to alternate incubation in a solution of 5 × 10^− 3^ M ethylenediaminetetraacetic acid (EDTA; Cat. No. E809068, Macklin) at pH 7.4 or collagenase II. The resulting bone particles were then incubated in α-MEM supplemented with 5% fetal bovine serum (FBS; Cat. No. 12,664,025, Gibco), 5% calf serum (CS; Cat. No. A3520502, Gibco), and 2% P/S. The primary osteocytes migrated out of the bone particles within 7 days, and subsequent experiments were performed using cells at passage 0.

Primary BMSCs and primary bone marrow macrophages/monocytes were extracted from bone marrow samples of 6-week-old male C57BL/6J mice following previously published protocols [5, 46, 47]. BMSCs were cultured in α-MEM supplemented with 10% FBS and 1% P/S, while primary bone marrow macrophages/monocytes were cultured in high glucose Dulbecco’s modified eagle medium (DMEM; Cat. No. PM150210, Procell) supplemented with 10% FBS and 1% P/S. Cells in passage 1 were used in all experiments.

All cells were maintained at 37°C in a 95% humidified atmosphere with 5% CO_2_.

### Isolation of osteocyte-derived EVs

To eliminate the possibility of EV contamination in FBS and CS, ultracentrifugation was applied to deplete EVs from both FBS and CS. The primary YO and SO cells were initially maintained for 7 days to establish the cultures. Subsequently, the culture medium was replaced with α-MEM supplemented with 5% exosome-depleted FBS, 5% exosome-depleted CS, and 1% P/S. The cells were cultured with fresh medium for 48 h, followed by a medium replacement. This process was continued over 14 days. To isolate osteocyte-derived EVs, the cell culture medium was then subjected to sequential centrifugation steps at 300 × g for 10 min, 2 000 × g for 30 min, and 10 000 × g for 30 min at 4 °C. The resulting supernatant was further filtered through a 0.22-µm filter (Cat. No. SLGP033RS, Millipore) to eliminate any remaining cellular debris. The EVs were subsequently pelleted through ultracentrifugation at 100 000 × g and 4 °C for 10 h using a Beckman Optima XPN ultracentrifuge. The EV pellets were resuspended in PBS and preserved at − 80 °C, with precautions taken to avoid multiple freeze−thaw cycles before use.

### Identification of YO-EVs and SO-EVs

The concentration of EVs was estimated based on the total protein content, determined using a bicinchoninic acid (BCA) protein assay kit (Cat. No. E-BC-K318-M, Elabscience). Nanoparticle tracking analysis (NTA) with a ZetaView PMX 110 (Particle Metrix) was used to analyze the distribution and size of EVs, while Hitachi H-7650 transmission electron microscope (TEM; Hitachi) was used to identify the morphologies of EVs. To assess the effectiveness and stability of each batch of EVs, cellular functional tests and NTA analysis were employed. Flow cytometry on a FACSCANTO II (BD Biosciences) was employed to resolve EV surface marker proteins, including CD63, CD81, and TSG101, as previously described [48, 49], and FlowJo software (Tree Star Inc) was used to analyze the results. The CD63 polyclonal antibody (Cat. No. 25682-1-AP, Proteintech), CD81 monoclonal antibody (Cat. No. SC7637, Santa Cruz Biotechnology), TSG101 polyclonal antibody (Cat. No. 14497-1-AP, Proteintech) were used as primary antibodies. Alexa Fluor 488-conjugated goat anti-rabbit IgG (Cat. No. 111-545-144, Jackson ImmunoResearch) and Alexa Fluor 594-conjugated donkey anti-mouse IgG (Cat. No. 715-585-151, Jackson ImmunoResearch) were used as secondary antibodies.

### Osteogenesis and adipogenesis differentiation assay

BMSCs were seeded at densities of 5.0 × 10^4^ cells or 1.0 × 10^5^ cells per well in 48-well plates. Then, the cells were cultured in either osteogenic induction medium (Cat. No. MUBMD-90,021, Cyagen Biosciences) or adipogenic induction medium (Cat. No. MUBMD-90,031, Cyagen Biosciences) to induce osteogenic or adipogenic differentiation, respectively. To evaluate the effects of different treatments, 50 µg/mL of YO-EVs, SO-EVs, EVs from YB-OC-CM, EVs from SB-OC-CM, YO^si−Control^-EVs, YO^si−Tpm1^-EVs, or 5 µL of concentrated osteoclast culture medium cocultured with young bone slices or senescent bone slices (YB-OC-CM or SB-OC-CM) were added to the above induction medium. During longer culture periods for the Alizarin Red S (ARS) and Oil Red O (ORO) staining, the culture medium was changed every 2 to 3 days. The solvent group (PBS-treated) was set as positive control to ensure that any observed effects were specifically due to the EVs. To assess the direct influence of tropomyosin 1 (TPM1) overexpression on osteoblastic and adipogenic differentiation, we constructed a lentiviral vector to express TPM1 under the EF1A promoter (Fig. S9A). This vector was used to transfect BMSCs, which were then subjected to osteoblastic or adipogenic differentiation protocols.

Two days after induction, the cells were collected and processed for quantitative real-time PCR (qRT−PCR) to examine the expression of osteogenic or adipogenic genes. Alkaline phosphatase (ALP) activity was measured by staining the cells with an ALP stain kit (Cat. No. 40749ES60, Yeasen) or using an ALP assay kit (Cat. No. A059-2-2, Nanjing Jiancheng) at 3 days after induction. In order to identify the presence of mineralized nodules or lipid droplets, the cells were subjected to staining with Alizarin Red S (ARS) solution (G1452; Solarbio) at 7 days following osteogenic induction, or Oil Red O (ORO) solution (G1262; Solarbio) at 15 days following adipogenic induction. F-actin polymerization was revealed by TRITC-labeled phalloidin (Cat. No. 40734ES75, Yeasen). The stained cells were photographed with an inverted microscope (DMI6000B, Leica). The quantification of ALP^+^, ARS^+^, and ORO^+^ areas was conducted using Image-Pro Plus 6 software.

### Osteoclastogenesis differentiation assay

Bone marrow macrophages/monocytes were seeded at a density of 1.0 × 10^4^ cells per well in 48-well plates. Then, the cells were cultured in complete medium supplemented with 100 ng mL^− 1^ receptor activator for nuclear factor κB ligand (RANKL; Cat. No. 315 − 11, Peprotech) as an osteoclastogenesis induction factor. To assess the effects of different treatments, 50 µg/mL of YO-EVs, or SO-EVs was added to the above induction medium. During longer culture periods for the tartrate-resistant acid phosphatase (TRAP) staining, the culture medium was changed every 2 to 3 days. Two days after induction, the cells were collected and subjected to qRT−PCR to examine the expression of osteoclastic genes. The formation of multi-nucleated and large-spread mature osteoclasts was detected by staining the cells with a tartrate-resistant acid phosphatase (TRAP) staining kit (Cat. No. 387 A, Sigma − Aldrich). TRAP^+^ cells with more than three nuclei were counted as osteoclasts and photographed with an inverted microscope (DMI6000B, Leica).

### Preparation of osteoclast resorption culture medium with bone slices

In order to prepare osteoclastic bone resorption culture medium (OC-CM), various culture plates were used to host young or senescent bone slices without periosteum or bone marrow. Following previous reports [16], primary macrophages/monocytes were seeded onto bone slices at a density of 1.0 × 10^4^ cells per well, and this mixture was cultured in osteoclastic induction medium for 7 days. This permitted the development of mature osteoclasts, which were capable of initiating bone resorption during this time. Afterward, fresh osteoclastic induction medium with EV-depleted FBS was introduced to the culture, replacing the older medium. This new medium was collected after two days to obtain the conditional culture media. To demonstrate the resorptive activity of osteoclasts on bone slices, we performed scanning electron microscopy (SEM; S-3400, Hitachi) analysis after a 14-day culture period to observe resorption lacunae on bone slices. Young bone slices, senescent bone slices, or a mixture without bone was used to create the respective conditional medium, YB-OC-CM, SB-OC-CM, or OC-CM. These different types of OC-CM were either concentrated 10-fold or underwent EV purification using ultracentrifugation. The concentrated OC-CM, as well as any collected EVs, were stored at − 80 °C until use.

### Inhibition of Tpm1

Mouse Tpm1 siRNA and control siRNA were procured from RiboBio. The specific siRNA serial numbers and sequences employed in this study included the following: si-m-Tpm1-001, siG2107140843153170, GAAGGAAGACAAATATGAA; si-m-Tpm1-002, siG2107140843154262, GACGTAGCTTCTCTGAACA; si-m-Tpm1-003, siG2107140843155354, CAAGCACATTGCTGAAGAT. For the transfection of osteocytes, a concentration of 50 µM siRNA was utilized, followed by the utilization of Lipofectamine 3000 transfection reagent (Cat. No. L3000008, Thermo Fisher), in agreement with the manufacturer’s protocol.

### Animals and treatments

C57BL/6 mice were purchased from Hunan SJA Laboratory Animal Co. Ltd. and were allowed to adapt to their environment for one week. In order to investigate the effects of YO-EVs and SO-EVs, 3-month-old or 15-month-old male C57BL/6 mice were randomly divided into three groups: solvent, YO-EVs, and SO-EVs, with *n* = 5 per group. To study the effects of Tpm1, animals were randomly divided into three groups: solvent, YO^si−Control^-EVs, and YO^si−Tpm1^-EVs, with *n* = 5 per group. The solvent control groups were treated with an equal volume of PBS. The intervention for these groups was conducted by tail vein injection. Four weeks after the treatment, bone and blood samples were collected from mice and processed for subsequent experiments.

### µCT analysis

All harvested right femora were fixed in 4% paraformaldehyde for 48 h before measurement by µCT scanning. A vivaCT80 scanner (SCANCO Medical AG) was utilized with a voltage of 50 kV, a current of 400 µA, and a resolution of 18 μm per pixel. After scanning, bone reconstruction and visualization were performed using CT Analyser 1.11.0.0, µCTVol 2.2.0.0, and Dataviewer 1.4.3. Trabecular parameters were assessed at the distal femoral metaphysis by defining a region of interest (ROI) starting from 0.15 mm below the distal epiphyseal growth plate and extending proximally for 0.4 mm, allowing for measurement of parameters, including trabecular bone volume fraction (Tb. BV/TV), trabecular bone thickness (Tb. Th), trabecular number (Tb. N), trabecular separation (Tb. Sp). Cortical parameters were measured in a region that constituted 5% of the femoral length at the femoral mid-diaphysis, with the cortical bone thickness (Ct. Th) being assessed.

### Cellular internalization of EVs

For the EV internalization experiments, YO-EVs and SO-EVs were labeled with 1,1’-dioctadecyl-3,3,3’,3’-tetramethylindocarbocyanine perchlorate (DiI; Cat. No. 40726ES10, Yeasen) according to the manufacturer’s instructions. Following the removal of redundant dye through utilization of an Amicon 0.5 mL Ultracentrifugal Filter equipped with a 100 kDa membrane (Cat. No. UFC510024, Millipore), the labeled EVs were subjected to a 3-hour incubation period with BMSCs at a temperature of 37 °C. Subsequently, the treated cells underwent a rinsing process with PBS and were subsequently fixed in a 4% paraformaldehyde solution for a duration of 15 min. The stained cells were then subjected to two rounds of PBS washing before being mounted on coverslips using a commercially available antifade mounting medium containing 4’,6-diamidino-2-phenylindole (DAPI; Cat. No. H-1200-10, Vectorlabs). The internalization of the red DiI-labeled EVs by the cells was observed using a Zeiss ApoTome fluorescence microscope.

### Ex vivo biodistribution of EVs

To assess the EV distribution in vivo, YO-EVs and SO-EVs were purified and labeled using 1,1-dioctadecyl-3,3,3,3-tetramethylindotricarbocyaine iodide (DiR; Cat. No. 40757ES25, Yeasen) lipophilic dye according to the manufacturer’s instructions. After a 30-minute incubation period, unincorporated DiR was eliminated using Amicon 0.5 mL Ultracentrifuge Filters equipped with 100 kDa membranes. To evaluate whether EVs could effectively reach the bone, equal amounts of DiR-labeled YO-EVs and DiR-labeled SO-EVs were injected intravenously via the tail vein. After a 12-hour interval, the bone specimens were retrieved and analyzed using a fluorescence tomography imaging system (Cat. No. FMT4000, PerkinElmer) to quantify the fluorescence intensity.

### EV tracer transgenic mice

*Cd63*^*loxp−mCherry−loxp−eGFP*^ EV tracer transgenic mice were created utilizing the CRISPR/Cas9 homologous recombination technique. Cas9 mRNA and gRNA were generated via in vitro transcription. To generate the *Cd63*^*loxp−mCherry−loxp−eGFP*^ EV tracer transgenic mice, a homologous recombination donor vector was constructed. This vector consisted of a 3.4 kb 5’ homologous arm, loxp-mCherry-loxp-eGFP, and a 4.0-kb 3’ homologous arm, which was inserted at the termination codon of the Cd63 gene. Fertilized eggs from C57BL/6J mice were then microinjected with Cas9 mRNA, gRNA, and the donor vector to produce the F0 generation mice. The identification of homologous recombinant F0 generation mice was accomplished through the utilization of long fragment PCR. *Dmp1*^*Cre*^;*Cd63*^*loxp−mCherry−loxp−eGFP*^ mice, which have a tracer specific to osteocyte-derived EVs, were obtained by breeding homogenously recombinant F0 generation mice with *Dmp1*^*Cre*^ mice. The genotyping of the *Dmp1*^*Cre*^;*Cd63*^*loxp−mCherry−loxp−eGFP*^ mice is shown in Supplementary Fig. S2. The genotyping analysis of hemizygous and wild-type mice was performed by PCR, with the *Dmp1*^*Cre*^ allele producing a 324 bp PCR product in hemizygous mice and a 167 bp PCR product in wild-type mice. Similarly, the *Cd63*^*loxp−mCherry−loxp−eGFP*^ allele produced a 754 bp PCR product in hemizygous mice and a 488 bp PCR product in wild-type mice.

Femora harvested from *Dmp1*^*Cre*^;*Cd63*^*loxp−mCherry−loxp−eGFP*^ mice were used for fluorescence detection of mCherry and eGFP signals using a fluorescence microscope. The eGFP antibody (Cat. No. ab290, Abcam) was used to stain eGFP.

### qRT−PCR analysis

Total RNA was extracted from cells, bone matrix, and bone marrow samples using TRIzol reagent (Cat. No. 15,596,026, Invitrogen), and cDNA was synthesized from 1.0 µg of total RNA using the Revert Aid First Strand cDNA synthesis kit (Cat. No. K1622, Thermo Scientific). The FTC-3000 real-time PCR system (Funglyn Biotech) was then used to perform quantitative real-time PCR (qRT−PCR) analysis using GoTaq® qPCR Master Mix (Cat. No. A6002, Promega). To assess the relative mRNA expression levels, the relative standard curve method (2^–△△CT^) with GAPDH as an mRNA normalization control was employed. The following primer sequences were used for qRT−PCR: *Dmp1*: forward, 5'-GAAAGCTCTGAAGAGAGGACGG-3', and reverse, 5'-CCTCTCCAGATTCACTGCTGTC-3'; *Col1a1*: forward, 5'-GCTCCTCTTAGGGGCCACT-3', and reverse, 5'-CCACGTCTCACCATTGGGG-3'; *P16*: forward, 5'-TGTTGAGGCTAGAGAGGATCTTG-3', and reverse, 5'-CGAATCTGCACCGTAGTTGAGC-3'; *P21*: forward, 5'-TCGCTGTCTTGCACTCTGGTGT-3', and reverse, 5'-CCAATCTGCGCTTGGAGTGATAG-3'; *Runx2*: forward, 5'-GACTGTGGTTACCGTCATGGC-3', and reverse, 5'-ACTTGGTTTTTCATAACAGCGGA-3'; *Pparg*: forward, 5'-TCGCTGATGCACTGCCTATG-3', and reverse, 5'-GAGAGGTCCACAGAGCTGATT-3'; *Ctsk*: forward, 5'-GCGGCATTACCAACAT-3', and reverse, 5'-CTGGAAGCACCAACGA-3'; *Tpm1*, forward, 5'-AACGGTGACGAACAACTTGAA-3', and reverse, 5'-GGAAGTCATATCGTTGAGAGCG-3'; and *Gapdh*: forward, 5'-CACCATGGAGAAGGCCGGGG-3', and reverse, 5'-GACGGACACATTGGGGGTAG-3'.

### Enzyme-linked immunosorbent assay (ELISA)

The serum levels of osteocalcin (OCN) and cross-linked C-telopeptide of type I collagen (CTX-I) were assessed using mouse ELISA kits E-EL-M0864c and E-EL-M3023 (Elabscience). All ELISA were conducted in accordance with the manufacturer’s instructions, and the protein concentration for each sample was calculated based on the standard curve.

### Histomorphometry and immunostaining

Freshly harvested femora were first fixed in 4% paraformaldehyde for 48 h prior to undergoing decalcification in 10% EDTA at pH 7.4 for 7 days. Following decalcification, samples were embedded in paraffin and sliced into longitudinally oriented sections with a thickness of 5 μm. Subsequently, the sections were subjected to staining for osteocalcin (OCN), perilipin (PLIN), and TRAP to identify osteoblasts, adipocytes, and osteoclasts, respectively. Appropriate secondary antibodies were then incubated, followed by countered with DAPI. Images were acquired using either an optical microscope (Olympus CX31) or a fluorescence microscope (Zeiss ApoTome). The abundance of osteoblasts and osteoclasts was quantified as the number per millimeter of the bone surface, denoted as N. OBs/BS/mm and N. OCs/BS/mm. Similarly, the number of adipocytes per square millimeter of marrow tissue was quantified and recorded as N. AdCs/Ar/mm^2^.

### Three-point bending test

The strength of the femur at the midshaft location was measured by subjecting bones to a three-point bending test on a mechanical-testing machine (Cat. No. 3343, Instron). This involved utilizing two end-support points and one central loading point, with the span length between the support points constituting 60% of the total bone length. Bones were subjected to a constant loading speed of 0.155 mm per second until failure occurred. The maximum load (N) was recorded using load−deformation curves obtained through biomechanical measurements.

### Proteomic analysis

YO-EVs and SO-EVs (*n* = 3 for each group) were subjected to label-free quantitative proteomic analysis, which was performed by Jingjie PTM BioLab. Using the criteria of YO-EVs/SO-EVs > 1.5 or < 0.67 and *P* < 0.05, differentially enriched proteins were identified. To determine the enrichment of functions, the Gene Ontology (GO) database was employed for analysis. The proteins that exhibited a YO-EVs/SO-EVs ratio greater than 1.5 were subjected to mapping onto Gene Ontology (GO) terms. Subsequently, the GO terms that showed significant enrichment were determined using a threshold of *P* < 0.05. These enriched GO terms were then categorized into three distinct groups: biological process (BP), cellular component (CC), and molecular function (MF).

### Statistical analysis

The results are expressed as the mean ± standard deviation (SD), and the sample size (n) for each statistical analysis is specified in the Fig. legends. The statistical significance of differences between various treatments was assessed using either the two-tailed Student’s t-test or one-way ANOVA with Bonferroni post-test. The data analyses were performed utilizing GraphPad Prism 9.1 software. A statistically significant level was set at *P* < 0.05, *P* < 0.01, *P* < 0.001, and *P* < 0.0001, which were denoted by “*”, “**”, “***”, and “****”, respectively.

## Results

### Stimulation of BMSC osteogenesis by YO-EVs and induction of BMSC adipogenesis by SO-EVs

**Fig. 1:**
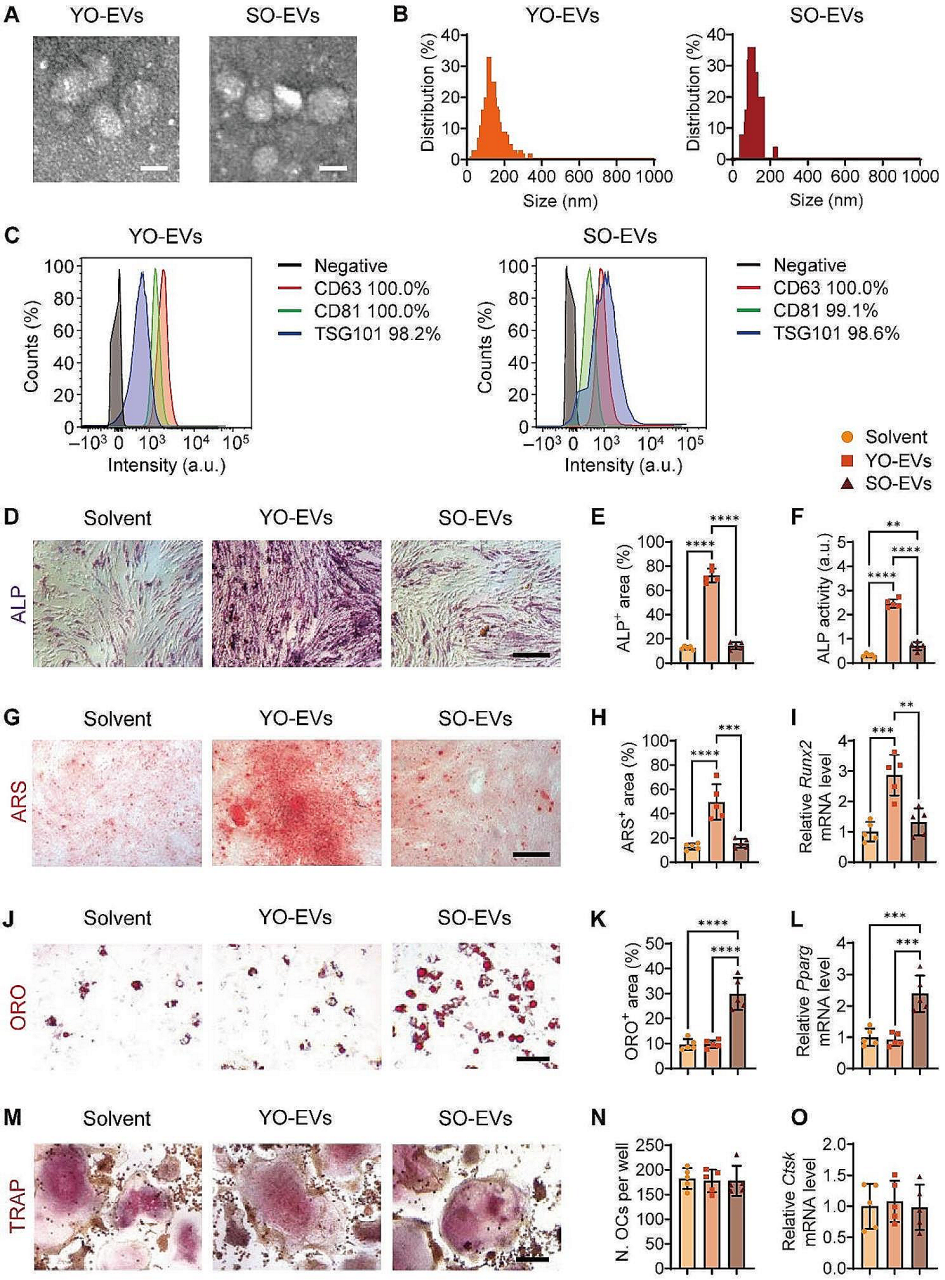
Stimulation of BMSC osteogenesis by YO-EVs and induction of BMSC adipogenesis by SO-EVs. Representative morphological analysis by TEM (**A**), diameter distribution by NTA (**B**), and classical EV markers (CD63, CD81, and TSG101) by flow cytometry (**C**) of YO-EVs and SO-EVs. Scale bar: 100 nm. Secondary antibody incubation alone served as the negative control (black curves). ALP staining (**D**; violet), ARS staining (**G**; red), quantification of the percentages of ALP^+^ area (**E**) and ARS^+^ area (**H**), cellular ALP activity (**F**), and qRT−PCR analysis of *Runx2* (**I**) of BMSCs treated with Solvent, YO-EVs, or SO-EVs under osteogenic induction. ORO staining (**J**; red), quantification of the percentages of ORO^+^ area (**K**), and qRT−PCR analysis of *Pparg* (**L**) of BMSCs treated with Solvent, YO-EVs, or SO-EVs under adipogenic induction. TRAP staining (**M**; violet), quantification of the number of osteoclasts (OCs) per well (**N**), and qRT−PCR analysis of *Ctsk* (**O**) of BMMs treated with Solvent, YO-EVs, or SO-EVs under osteoclastogenic induction. Scale bar: 50 μm. *n* = 5 per group. PBS treatment was used in the solvent groups. ** *P* < 0.01, *** *P* < 0.001, **** *P* < 0.0001

In this study, primary young osteocytes (YO) and senescent osteocytes (SO) were isolated from C57BL/6 mice aged 2 and 16 months, respectively. The gene expression analyses were performed to detect marker genes associated with osteocytes and senescence. The osteocyte-specific marker gene dentin matrix acidic phosphoprotein 1 (*Dmp1*) and the osteoblast-specific marker gene collagen type I alpha 1 chain (*Col1a1*) were analyzed to confirm the osteocytic nature of YO and SO (Supplementary Fig. S1A). Furthermore, to ascertain the senescent status, we evaluated the expression levels of senescence markers *P16* and *P21*. Our results showed that SO exhibited significantly higher expression levels of *P16* and *P21* than YO, confirming their senescent status (Supplementary Fig. S1B). Subsequently, the cell culture medium was collected multiple times in order to obtain YO-derived extracellular vesicles (YO-EVs) and SO-derived EVs (SO-EVs) through ultracentrifugation. Consistent with previous reports [27], the isolated EVs displayed cup-shaped morphologies with diameters of 117.5 ± 32.5 nm (YO-EVs) or 107.5 ± 40.5 nm (SO-EVs), as revealed by transmission electron microscopy (TEM; Fig. 1A) and nanoparticle tracking analysis (NTA; Fig. 1B). Flow cytometry analysis in Fig. 1C revealed that the isolated EVs exhibited classical EV markers, such as CD63 (100% positive for YO-EVs and SO-EVs), CD81 (100% and 99.1% positive for YO-EVs and SO-EVs), and TSG101 (98.2% and 98.6% positive for YO-EVs and SO-EVs). These results confirmed that the vesicles obtained from osteocytes were EVs.

To evaluate the impact of YO-EVs and SO-EVs on the differentiation of BMSCs into osteogenic and adipogenic lineages, an initial investigation was conducted to determine the internalization potential of these EVs by primary mouse BMSCs. To facilitate this assessment, DiI, a red lipophilic dye, was employed to label YO-EVs and SO-EVs, followed by the removal of any excess dye through ultrafiltration. Next, BMSCs were exposed to DiI-labeled YO-EVs or SO-EVs for 3 h, and the unbound EVs were subsequently washed away. Confocal microscopy imaging revealed numerous red fluorescent signals in the perinuclear area of BMSCs (Supplementary Fig. S2), indicating the successful internalization of these EVs by BMSCs.

We then explored the potential of YO-EVs and SO-EVs to modulate the differentiation fate of BMSCs. When subjected to osteogenic induction, YO-EVs, but not SO-EVs, significantly increased the intensity of ALP staining (Fig. 1D, E) and ALP activity in the culture medium (Fig. 1F), indicating an enhancement in the osteogenic activity of BMSCs. Consistently, Alizarin Red S (ARS) staining intensity (Fig. 1G, H) and quantitative real-time PCR (qRT−PCR) analysis (Fig. 1I) showed that YO-EVs, but not SO-EVs, strongly promoted the calcium nodule formation of BMSCs and upregulated the expression of runt-related transcription factor 2 (*Runx2*), an osteogenesis-related gene, during osteogenic induction. In contrast, SO-EVs, but not YO-EVs, substantially increased lipid droplet formation and upregulated the expression of peroxisome proliferator-activated receptor gamma (*Pparg*), an adipogenesis-related gene, during adipogenic differentiation of BMSCs, as evidenced by Oil Red O (ORO) staining (Fig. 1J, K) and qRT−PCR analysis (Fig. 1L). Furthermore, the effects of YO-EVs and SO-EVs on osteoclastic differentiation were also assessed by inducing primary bone marrow macrophages/monocytes (BMMs) with RANKL. The intensity of tartrate-resistant acid phosphatase (TRAP) staining and qRT−PCR analysis revealed that neither YO-EVs nor SO-EVs had an obvious effect on osteoclast fusion (Fig. 1M, N) or the expression of cathepsin K (*Ctsk*; Fig. 1O), an osteoclastogenesis-related gene, during osteoclastic induction.

Taken together, these results indicated that while YO-EVs promoted BMSC osteogenesis, they lost this potency with aging and instead exhibited pro-adipogenic effects on BMSCs.

### Effects of age-related EVs released from bone matrix on BMSC differentiation

To investigate the presence of osteocyte-derived EVs in the cortical bone, we constructed EV tracer transgenic mice to visualize EV distribution. The CD63 protein, a membrane protein that is widely present in EVs and serves as a universal marker for practically all EVs, was genetically modified to construct transgenic mice. Specifically, homologous recombination CRISPR/Cas9 technology was employed to knock in the expression frame of “loxp-mCherry-loxp-eGFP” to the termination codon of the Cd63 gene, thus creating *Cd63*^*loxp−mCherry−loxp−eGFP*^ EV tracer mice. These mice exhibited constitutive expression of mCherry fusion protein in all cells. Upon mating with Cre recombinase tool mice, the loxp-mCherry-loxp sequence was excised, leading to conditional expression of the eGFP fusion protein in specific cells, allowing us to track certain cell-released EV localization. The murine dentin matrix acidic phosphoprotein 1 (Dmp1) promoter has been widely recognized as the most commonly used marker for identifying osteocytes. To enable targeted mutagenesis of osteocytes, we employed *Dmp1*^*Cre*^ mice, which express Cre recombinase under the direction of the mouse Dmp1 promoter. To generate CD63 conditional mutants, *Dmp1*^*Cre*^ mice were crossbred with *Cd63*^*loxp−mCherry−loxp−eGFP*^ mice. Genotyping of the resultant *Dmp1*^*Cre*^;*Cd63*^*loxp−mCherry−loxp−eGFP*^ mice was conducted and depicted in Supplementary Fig. S3.

**Fig. 2:**
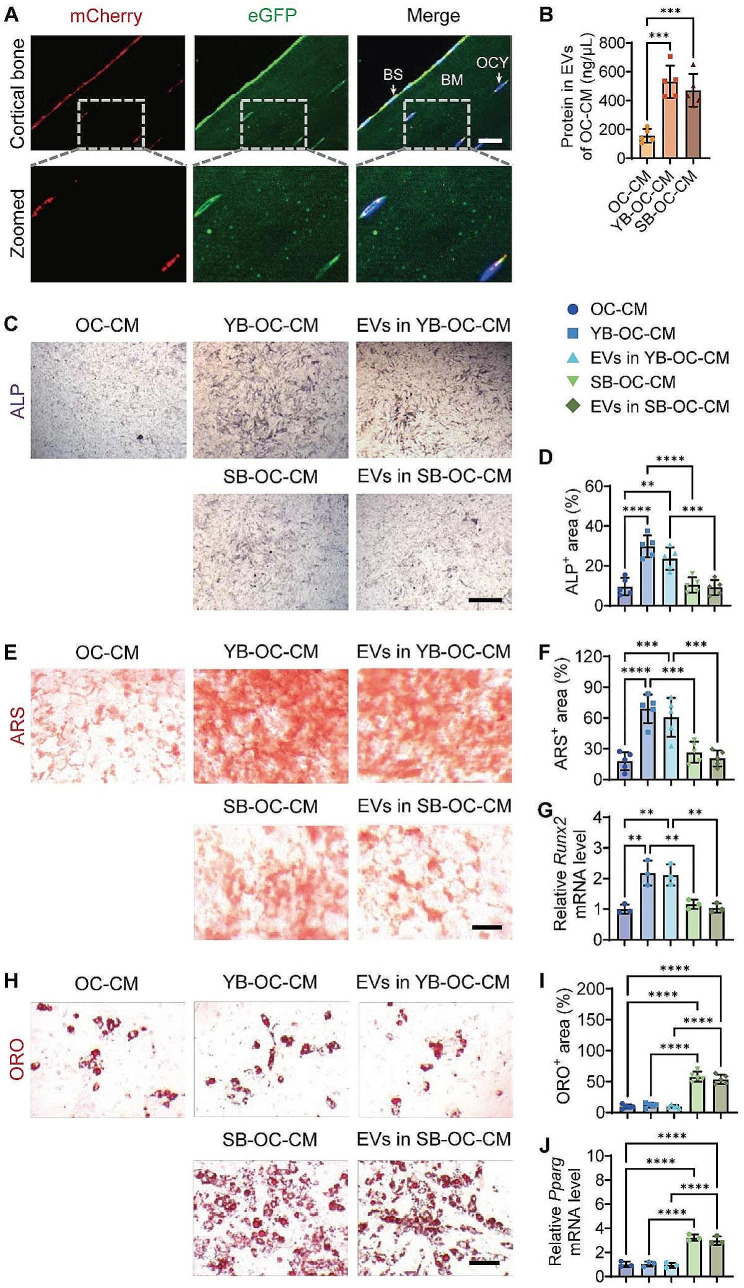
Effects of age-related EVs released from bone matrix on BMSC differentiation. Representative IF staining of mCherry (red) and eGFP (green) in bone matrix sections from *Dmp1*^*Cre*^;*Cd63*^*loxp−mCherry−loxp−eGFP*^ mice (**A**). Cell nucleus was stained with DAPI (blue). The merged images were overlaid on the right side, and the dashed square line indicated the zoomed regions. BS: bone surface, BM: bone matrix, OCY: osteocytes. Scale bar: 20 μm. Total protein contents of EVs isolated from the conditioned medium of mature osteoclasts treated with solvent (OC-CM), young bone (YB-OC-CM), or senescent bone (SB-OC-CM) (**B**). OC: osteoclasts; CM: conditioned medium. *n* = 5 per group. ALP staining (**C**; violet), ARS staining (**E**; red), ORO staining (**H**; red), quantification of the percentages of ALP^+^ area (**D**), ARS^+^ area (**F**), ORO^+^ area (**I**), qRT−PCR analysis of *Runx2* (**G**) and *Pparg* (**J**) of BMSCs with the indicated treatment under osteogenic or adipogenic induction. Scale bar: 50 μm. *n* = 5 or 3 (qRT−PCR analysis) per group. ** *P* < 0.01, *** *P* < 0.001, **** *P* < 0.0001

Cortical bone sections from *Dmp1*^*Cre*^;*Cd63*^*loxp−mCherry−loxp−eGFP*^ mice were prepared to observe the fluorescence signals of mCherry and eGFP. As presented in Fig. 2A, significant expression of mCherry red fluorescence was revealed on the bone surface, originating primarily from osteoblasts and osteoclasts, while comparatively little mCherry signal was detected in the perinuclear area of the osteocytes located within the cortical bone matrix. Interestingly, numerous dot-like eGFP green signals were detected within the bone matrix, indicating that the vast majority of these signals originate from osteocyte-derived EVs that naturally penetrate the bone matrix under physiological conditions.

The content in the bone matrix was mostly released by osteoclast resorption, with a minor contribution via the bone matrix microtubule system. The primary osteoclast progenitor, bone marrow macrophage/monocytes, was activated by receptor activator of nuclear factor-kappaB ligand (RANKL) and cultured with or without bone slices from young or senescent mice to obtain corresponding conditioned medium (YB-OC-CM or SB-OC-CM). To demonstrate the resorptive activity of osteoclasts on bone slices, we performed scanning electron microscopy (SEM) analysis after a 14-day culture period. The SEM images provide clear evidence of resorption lacunae on bone slices exposed to osteoclast cultures; in contrast, the control bone slices, which did not undergo osteoclastic induction, showed no such lacunae (Supplementary Fig. S4). To mimic osteocyte-derived EV release from the bone matrix, the CM from osteoclasts under various conditions was then extracted to analyze the changes in EV amounts and their effects on BMSC osteogenic and adipogenic differentiation. Because EV protein concentration is often regarded as a measure of EV abundance, the EV protein levels in various CMs were compared. As shown in Fig. 2B, osteoclast coculture with bone slices greatly increased EV generation in the CM compared to osteoclasts alone. However, there was no significant difference in EV protein levels between young and senescent bone slices, as evidenced by comparable amounts of EV proteins in the YB-OC-CM and SB-OC-CM groups.

In accordance with the observed impacts of isolated YO-EVs and SO-EVs on the differentiation of BMSCs, the application of YB-OC-CM was found to significantly enhance ALP activity (Fig. 2C-D), the formation of calcium nodules (Fig. 2E-F), and the expression of *Runx2* (Fig. 2G) in BMSCs during osteogenic induction. Whereas, SB-OC-CM did not exhibit any noticeable influence on these parameters (Fig. 2C-G). Conversely, ORO staining (Fig. 2H-I) and *Pparg* expression analysis (Fig. 2J) indicated a marked influence of SB-OC-CM on BMSC adipogenesis compared to OC-CM and YB-OC-CM. Furthermore, the effects of EVs extracted from YB-OC-CM (EVs in YB-OC-CM) and SB-OC-CM (EVs in SB-OC-CM) on BMSC differentiation were also studied. Similar to the corresponding CM, EVs in YB-OC-CM and SB-OC-CM separately enhanced osteogenic or adipogenic differentiation of BMSCs (Fig. 2C-J), underscoring the indispensable role of EVs in OC-CM from bone slices. These findings implied that during bone resorption, osteocyte-derived EVs might be released from the bone matrix and influenced BMSC differentiation fate.

### Effects of YO-EVs and SO-EVs on bone mass in young and elderly mice

The potent anabolic effects of EVs in YB-OC-CM on BMSC osteogenesis, together with the comparable activities of YO-EVs isolated from primary young osteocytes, prompted us to investigate their potential roles in stimulating bone formation in vivo. To determine whether osteocyte-derived EVs could reach bone tissue under physiological settings, YO-EVs and SO-EVs were tagged with 1,1’-dioctadecyl-3,3,3’,3’-tetramethylindotricarbocyanine iodide (DiR) near-infrared fluorescent dye and intravenously administered to 3-month-old wild type (WT) mice, followed by monitoring of EV distribution in bone tissue using a spectrum in vivo imaging system. Ex vivo fluorescence imaging revealed robust fluorescence signals in bone tissues at 24 h after injection, implying that both YO-EVs and SO-EVs could effectively penetrate into the bone tissue of WT mice (Supplementary Fig. S5).

**Fig. 3:**
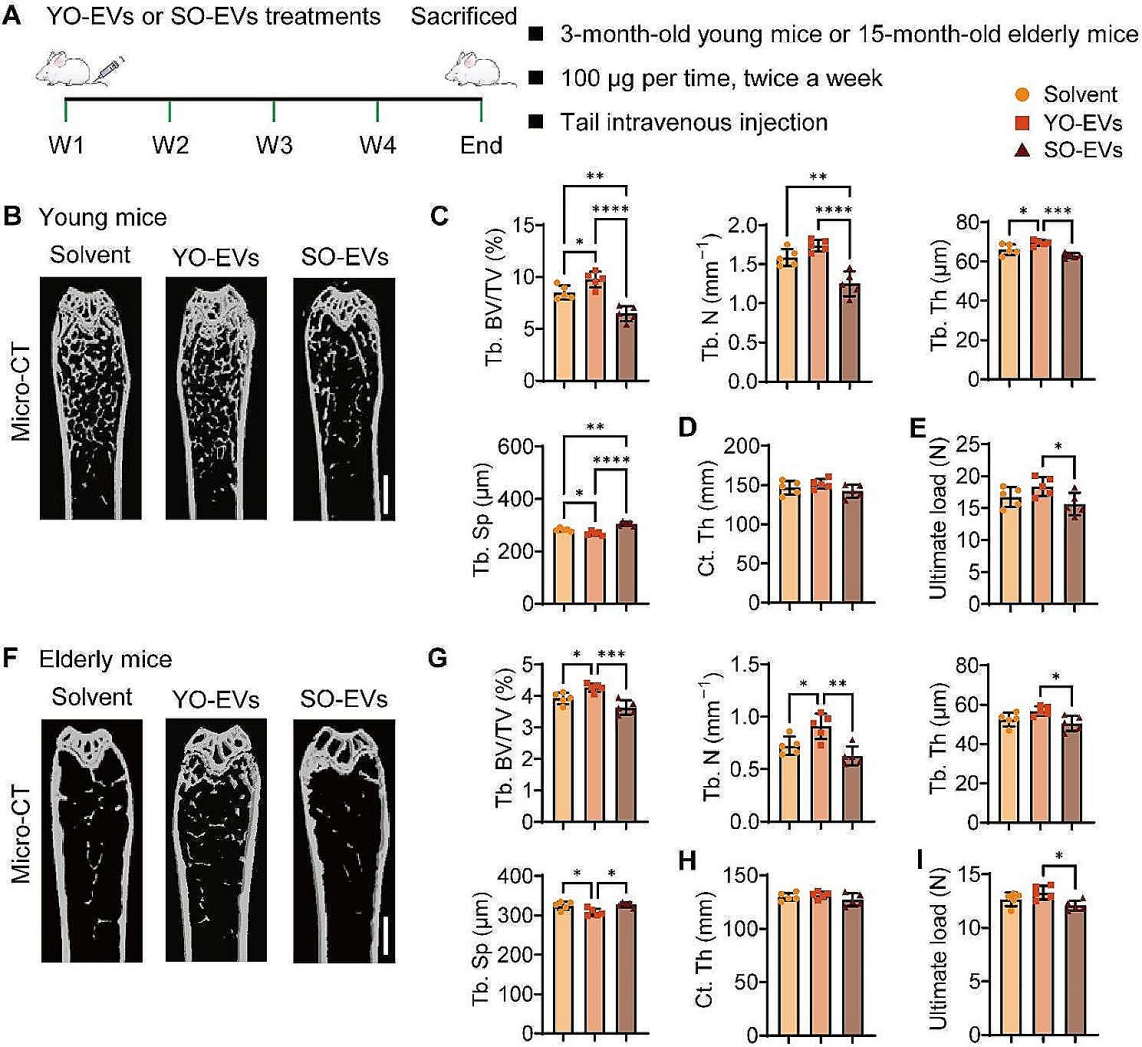
Effects of YO-EVs and SO-EVs on bone mass in young and elderly mice. Schematic diagram of the experimental design for assessing the effects of YO-EVs and SO-EVs on bone mass in young and elderly mice (**A**). µCT-reconstructed images of femurs from young mice (**B**) or elderly mice (**F**) treated with Solvent, YO-EVs, or SO-EVs. Scale bars: 1 mm. Quantification of Tb. BV/TV, Tb. N, Tb. Th, Tb. Sp of trabecular bone (**C** for young mice; **G** for elderly mice), and Ct. Th of cortical bone (**D** for young mice; **H** for elderly mice). Three-point bending measurement of the femoral ultimate load values from young mice (**E**) or elderly mice (**I**) with the indicated treatment. *n* = 5 per group. PBS treatment was used in the solvent groups. * *P* < 0.05, ** *P* < 0.01, *** *P* < 0.001, **** *P* < 0.0001

Next, we aimed to investigate whether administering YO-EVs via intravenous injection twice a week for a month could accelerate osteogenesis in 3-month-old young mice and mitigate age-related bone loss in 15-month-old elderly mice (Fig. 3A). The effects of SO-EVs on bone were also assessed in parallel. The microcomputed tomography (µCT) reconstructed images of femurs in both young and elderly mice revealed that YO-EV-treated mice had increased bone mass, whereas SO-EV-treated mice had reduced bone mass compared to the control group administered with solvent, as depicted in Fig. 3B and F. Notably, YO-EV-treated young and elderly mice exhibited significantly higher trabecular bone mass characteristics, as denoted by significantly elevated trabecular bone volume fraction (Tb. BV/TV), trabecular number (Tb. N), or/and trabecular thickness (Tb. Th), as well as reduced trabecular separation (Tb. Sp) compared to the corresponding control groups. In contrast, the administration of SO-EVs resulted in a significant reduction in Tb. BV/TV and Tb. N, coupled with an increase in Tb. Sp in young mice (Fig. 3C); the same trend was also observed in elderly mice, with SO-EVs causing a decrease in Tb. BV/TV and Tb. N (Fig. 3G). However, neither YO-EVs nor SO-EVs exhibited significant variations in cortical thickness (Ct. Th) in both the young and elderly groups (Fig. 3D and H). Furthermore, the three-point bending test demonstrated a positive impact of YO-EVs on femur ultimate load value in both young and elderly mice, while AB-EVs caused a decrease in this parameter in both groups (Fig. 3E and I).

These results suggested that YO-EVs might have a beneficial effect on bone strength, while SO-EVs may have a harmful effect.

### Enhancement of bone formation by YO-EVs and promotion of fat accumulation by SO-EVs in bone marrow

**Fig. 4:**
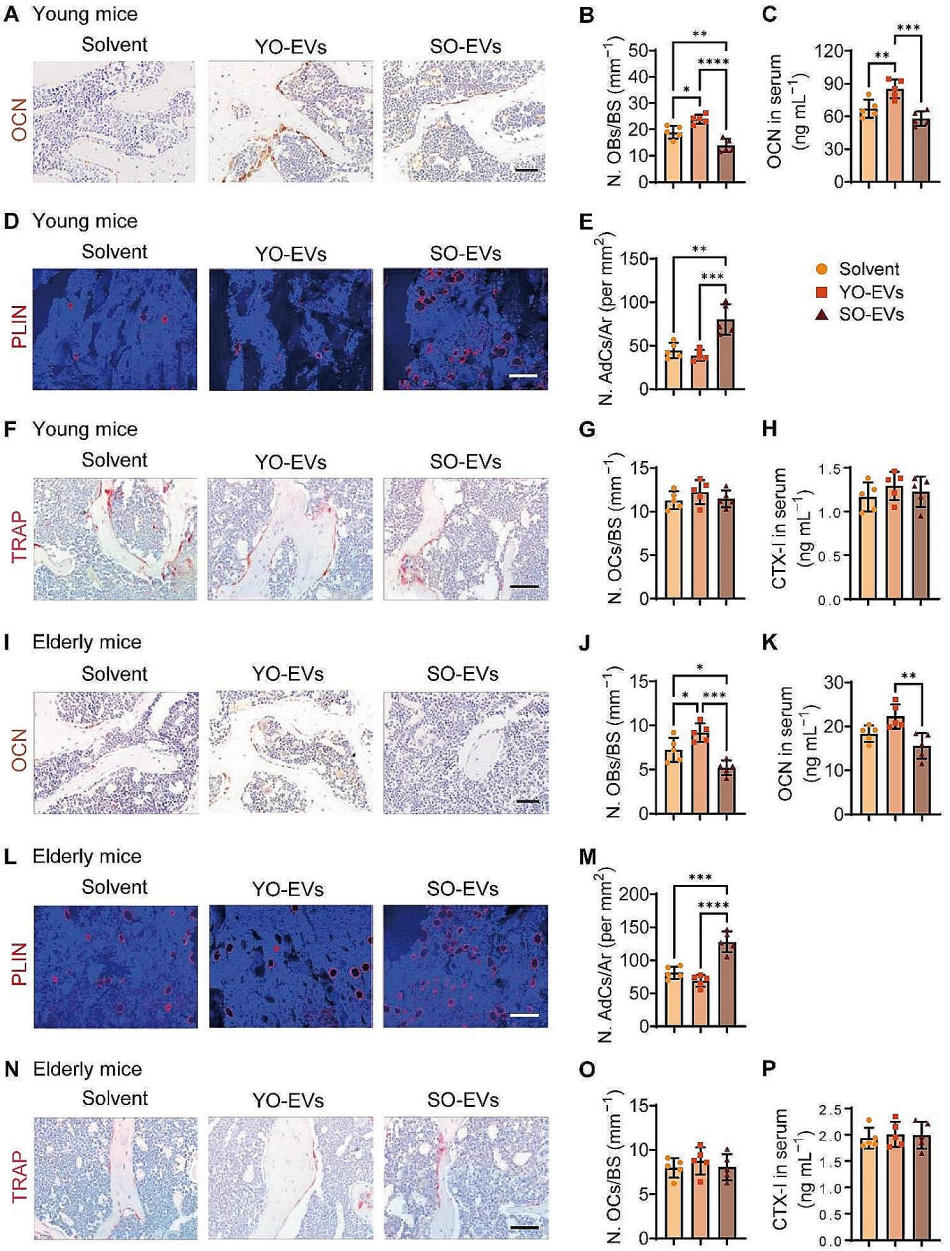
Enhancement of bone formation by YO-EVs and promotion of fat accumulation by SO-EVs in bone marrow. OCN immunohistochemical staining images (**A** for young mice; I for elderly mice), the number of OCN^+^ (brown) osteoblasts (N. OBs) on the trabecular bone surface (**B** for young mice; J for elderly mice), and the concentration of OCN in serum (**C** for young mice; K for elderly mice). PLIN immunofluorescence staining images (**D** for young mice; L for elderly mice), the number of PLIN^+^ (red) adipocytes in bone marrow (**N**. AdCs per mm^2^; **E** for young mice; M for elderly mice). TRAP histochemical staining images (**F** for young mice; **N** for elderly mice), the number of TRAP^+^ (red) osteoclasts (N. OCs) on the trabecular bone surface (**G** for young mice; **O** for elderly mice), and the concentration of CTX-I in serum (**H** for young mice; **P** for elderly mice). Scale bar: 50 μm (in **A, F, I**, and **N**) or 100 μm (in **D** and **L**). *n* = 5 per group. BS: bone surface, Ar: area. PBS treatment was used in the solvent groups. * *P* < 0.05, ** *P* < 0.01, *** *P* < 0.001, **** *P* < 0.0001

Furthermore, the femur tissues and blood samples from mice treated with solvent, YO-EVs, or SO-EVs were collected to evaluate osteoblastic, adipogenic, and osteoclastic activity. To assess the osteoblastic activity, immunohistochemistry staining for osteocalcin (OCN) was performed. The results showed a significant increase in osteoblast quantity on the bone surface of trabecular bones in both young (Fig. 4A-B) and elderly mice (Fig. 4I-J) treated with YO-EVs. Conversely, SO-EVs resulted in a significant reduction in the number of osteoblasts compared to the group treated with the solvent (Fig. 4A-B and I-J). Additionally, the levels of serum OCN were assessed by enzyme-linked immunosorbent assay (ELISA), and the results confirmed the beneficial impact of YO-EVs on osteogenic activity in young mice. However, the treatment of both young and elderly mice with SO-EVs showed a tendency towards decreased serum OCN levels compared to the control mice treated with the solvent (Fig. 4C). These findings suggested that YO-EVs participated in bone formation throughout adolescence but lost these capabilities during skeletal aging.

With advancing age, BMSCs undergo a shift in differentiation fate toward adipogenesis instead of osteogenesis. This phenomenon leads to the accumulation of adipocytes in bone tissue, which is commonly observed as “yellow bone marrow” instead of “red bone marrow” found in young mice. To assess the adipogenic activity, immunofluorescence staining of perilipin (PLIN), a protein found in adipocytes, was performed. Solvent-treated groups demonstrated a modest number of adipocytes in the bone marrow of young mice (Fig. 4D-E), whereas an increase in the adipocyte population was observed in elderly mice (Fig. 4L-M). Notably, the application of SO-EVs significantly increased the number of adipocytes in both the young and elderly groups compared to the solvent-treated groups (Fig. 4D-E and L-M). Conversely, the administration of YO-EVs tended to decrease the number of marrow adipocytes in both the young and elderly groups (Fig. 4D-E and L-M), likely due to the preference of YO-EVs for osteogenic differentiation over adipogenic differentiation of BMSCs.

Given the continuous bone formation and resorption process, we also investigated the effects of osteocyte-derived EVs on osteoclastic activity. Specifically, we examined the impact of YO-EVs and SO-EVs on osteoclast formation and activity in both young and elderly mice. Tartrate-resistant acid phosphatase (TRAP) staining (Fig. 4F-G and N-O) and ELISA analyses of C-terminal telopeptides of type I collagen (CTX-I) in serum (Fig. 4H and P) showed that neither YO-EVs nor SO-EVs significantly affected osteoclast formation and activity in either age group.

These findings indicated that YO-EV intervention might improve bone formation, but that this beneficial effect was lost with aging. Additionally, our results revealed that osteocyte-derived EVs did not affect osteoclastogenesis. Further elucidation of the underlying mechanisms of these complex processes is needed for a better understanding of bone metabolism.

### Proteomic profiling of YO-EVs and SO-EVs for comparative analysis

**Fig. 5:**
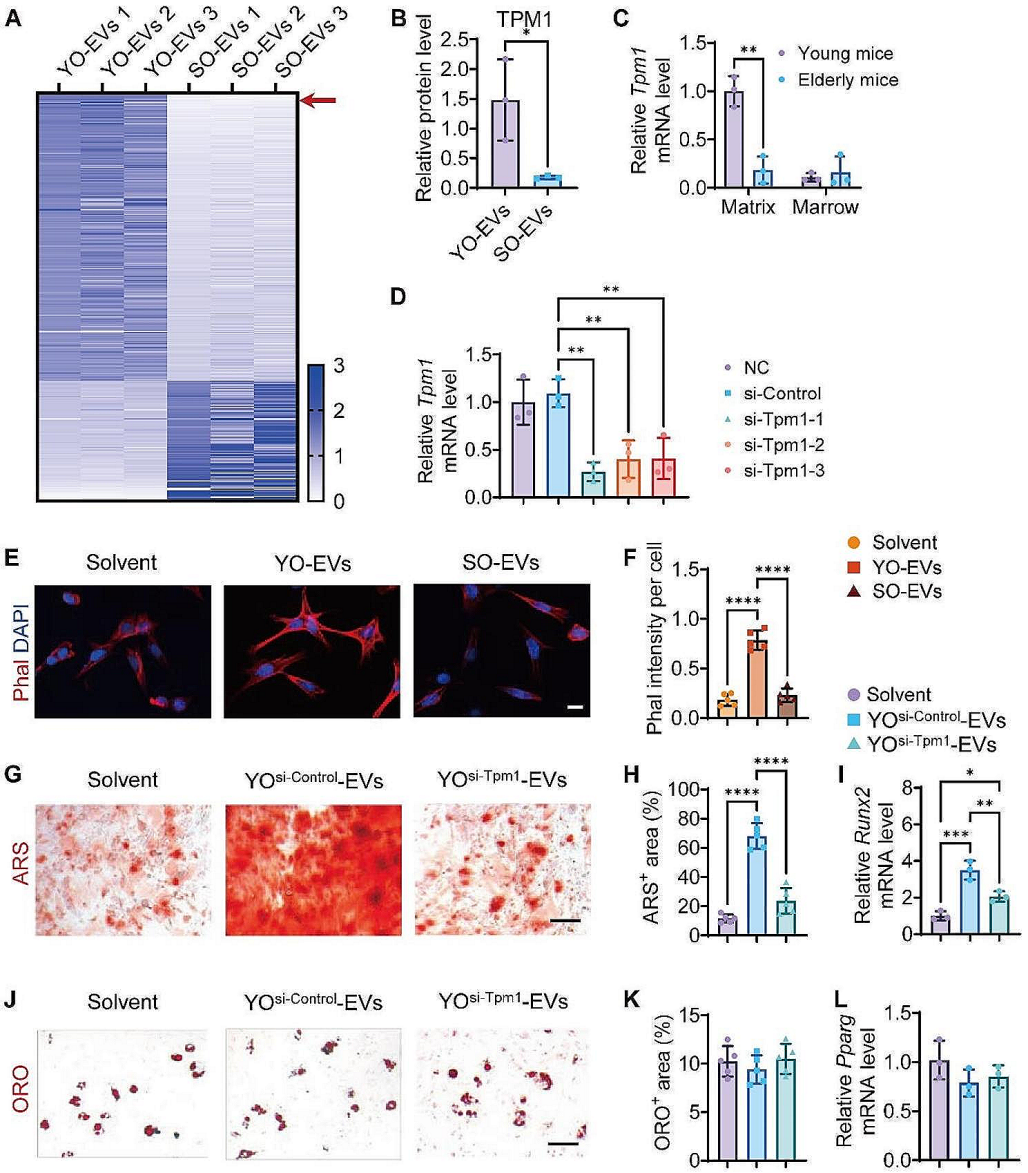
Proteomic profiling of YO-EVs and SO-EVs for comparative analysis. Heatmap showing the differentially enriched proteins (absolute fold change ≥ 1.5, *P* < 0.05) between YO-EVs and SO-EVs (**A**). *n* = 3 per group. Relative protein level of TPM1 in YO-EVs and SO-EVs (**B**). *n* = 3 per group. qRT−PCR analysis of *Tpm1* expression levels in bone matrix and bone marrow samples from young and elderly mice, respectively (**C**). *n* = 3 per group. The inhibitory efficiency of siRNAs targeting *Tpm1* was verified by qRT−PCR analysis (**D**). *n* = 3 per group. Phalloidin (Phal) immunofluorescence staining images (**E**) and the intensity of the Phal^+^ area (**F**) of BMSCs treated with Solvent, YO-EVs, or SO-EVs. Scale bar: 20 μm. *n* = 5 per group. ARS staining (**G**; red), ORO staining (**J**; red), quantification of the percentages of ARS^+^ area (**H**), ORO^+^ area (**K**), qRT−PCR analysis of *Runx2* (**I**) and *Pparg* (**L**) of BMSCs treated with Solvent, YO^si−Control^-EVs, or YO^si−Tpm1^-EVs under osteogenic or adipogenic induction. Scale bar: 50 μm. *n* = 5 or 3 (qRT−PCR analysis) per group. PBS treatment was used in the solvent groups. * *P* < 0.05, ** *P* < 0.01, **** *P* < 0.0001

Anile osteoporosis is primarily attributed to alterations in the microenvironment of bone tissue, wherein EVs play a significant role as modulators of intercellular communication. Notably, YO-EVs were found to promote greater osteogenic differentiation of BMSCs than SO-EVs. In order to elucidate the underlying mechanism of the pro-osteogenic effects of YO-EVs, we conducted a comprehensive analysis of the protein contents loaded onto YO-EVs and AO-EVs using a combined strategy involving TMT labeling and LC−MS/MS. Our analysis revealed the presence of 436 distinct proteins showing enhanced expression (greater than 1.5-fold, *P* 0.05) between YO-EVs and SO-EVs (Fig. 5A), with 335 and 101 proteins significantly expressed in YO-EVs and SO-EVs, respectively (Fig. 5A). Furthermore, gene ontology (GO) analyses were performed to gain insights into the biological context and associated biological processes. Among the differentially enriched proteins in YO-EVs, it was found that 83 molecular function (MF) terms, 135 biological process (BP) terms, and 104 cell component (CC) terms displayed considerable enrichment, with a difference greater than 1.5-fold (Supplementary Fig. S6-8). Based on the results shown in Supplementary Fig. S6, it was observed that YO-EVs contained a higher proportion of cytoskeleton control-related proteins, with the most enriched keywords of the molecular function being “actin filament binding”, “motor activity”, “microfilament motor activity”, and “actin-dependent ATPase activity”. Furthermore, tropomyosin 1 (TPM1) was identified as the most abundant cytoskeleton regulation-related protein in YO-EVs, but its expression decreased by approximately 8.24-fold in SO-EVs (Fig. 5B).

Quantitative real-time PCR (qRT−PCR) analysis was conducted to measure the alterations in Tpm1 gene expression in bone matrix and bone marrow samples obtained from 3-month-old young mice and 15-month-old senile mice. Figure 5C depicted the noticeable decline in Tpm1 expression in bone matrix with increasing age, while no significant variation was observed in bone marrow samples. The direct influence of TPM1 overexpression on osteoblastic and adipogenic differentiation was assessed by constructing a lentiviral vector to express TPM1 (Fig. S9A). After infection, these BMSCs were subjected to osteoblastic or adipogenic differentiation protocols. Our results showed that overexpression of TPM1 in BMSCs led to a significant increase in osteoblastic differentiation, as evidenced by enhanced ARS staining (Fig. S9B-C). In contrast, adipogenic differentiation, as indicated by ORO staining, was reduced in BMSCs overexpressing TPM1 (Fig. S9D-E). For the purpose of validating the essential role of TPM1 in YO-EVs, osteocytes were subjected to TPM1 depletion, and the resulting silencing effects were authenticated by conducting qRT−PCR analysis. Among the three synthesized candidate-specific small interfering RNA (siRNA) molecules targeting Tpm1, si-Tpm1-1 demonstrated the most effective inhibition and was therefore selected for further investigations (Fig. 5D).

Previous studies have demonstrated the role of TPM1 in facilitating the osteogenic differentiation of BMSCs by enhancing the stability of actin stress fibers [28]. In this study, we investigated the effect of YO-EVs and SO-EVs on the polymeric fibrous actin (F-actin) polymerization of BMSCs during osteogenic induction. F-actin staining demonstrated that YO-EVs, which were highly enriched with TPM1, intensified F-actin polymerization, whereas SO-EVs, which comprised low levels of TPM1, led to less F-actin polymerization (Fig. 5E-F). To validate the crucial role of TPM1 in regulating BMSC differentiation fate, we contrasted the effects of EVs from si-Control-treated young osteocytes (YO^si−Control^-EVs) or si-Tpm1-treated young osteocytes (YO^si−Tpm1^-EVs). ARS staining (Fig. 5G-H) and *Runx2* expression analysis (Fig. 5I) revealed that BMSCs treated with YO^si−Tpm1^-EVs generated significantly reduced mineralized nodule formation compared with those treated with YO^si−Control^-EVs. Additionally, ORO staining (Fig. 5J-K) and *Pparg* expression analysis (Fig. 5L) indicated that YO^si−Tpm1^-EVs did not significantly improve the capacity of YO-EVs to affect lipid droplet production in mouse BMSCs during adipogenesis.

These findings suggested that TPM1 acted as a critical mediator of YO-EV-induced osteogenic differentiation of BMSCs, most likely through the modulation of F-actin polymerization.

### Contribution of TPM1 to YO-EV-promoted bone formation

**Fig. 6:**
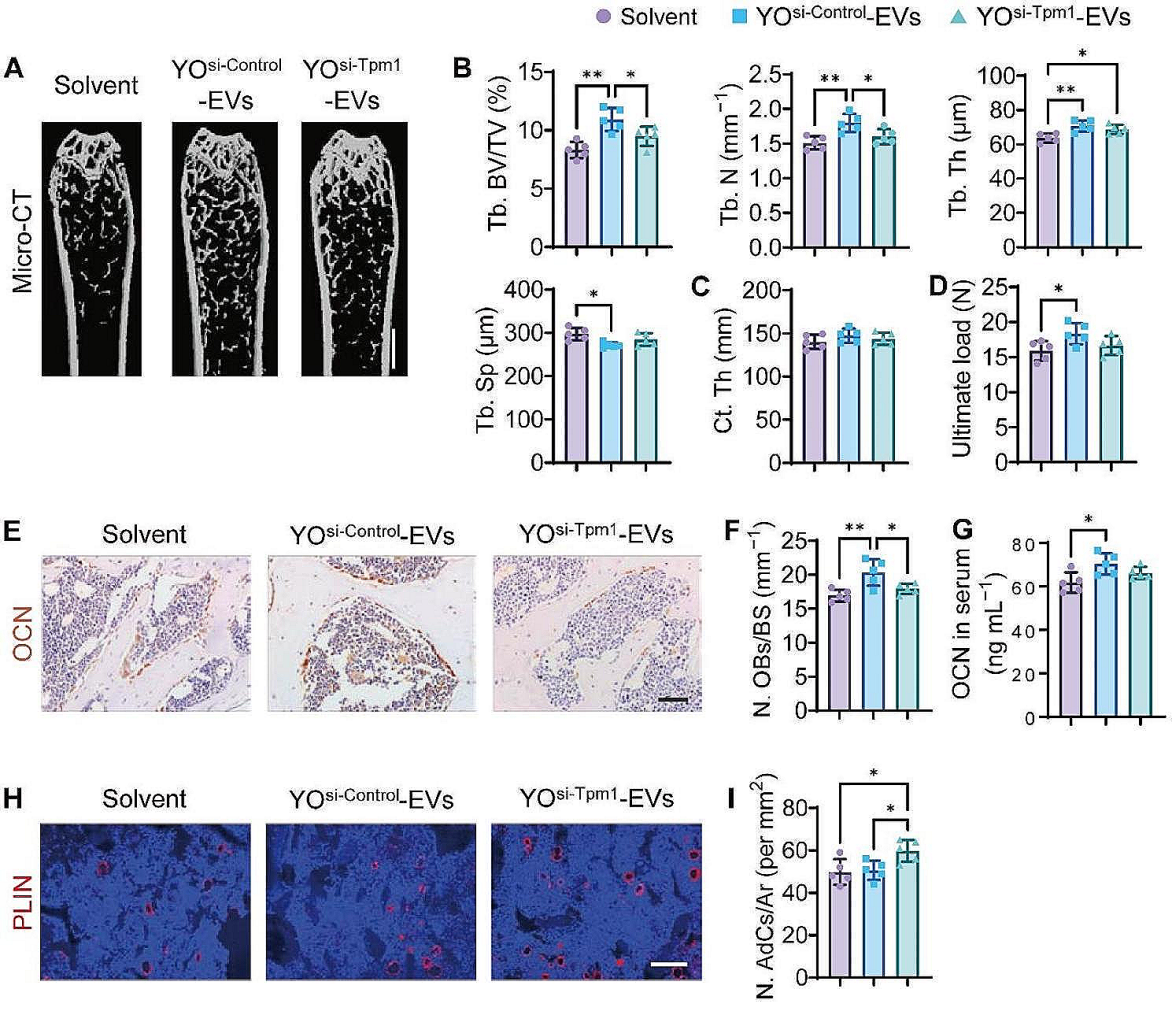
Contribution of TPM1 to YO-EV-promoted bone formation. µCT-reconstructed images of femurs from mice treated with Solvent, YO^si−Control^-EVs, or YO^si−Tpm1^-EVs (**A**). Scale bar: 1 mm. Quantification of Tb. BV/TV, Tb. N, Tb. Th, Tb. Sp of trabecular bone (**B**), and Ct. Th of cortical bone (**C**). Three-point bending measurement of the femoral ultimate load values from different treated mice (**D**). OCN immunohistochemical staining images (**E**), the number of OCN^+^ (brown) osteoblasts (**N**. OBs) on the trabecular bone surface (**F**), and the concentration of OCN in serum (**G**). PLIN immunofluorescence staining images (**H**), the number of PLIN^+^ (red) adipocytes in bone marrow (N. AdCs per mm^2^; I). Scale bar: 50 μm (in E) or 100 μm (in **H**). *n* = 5 per group. BS: bone surface, Ar: area. PBS treatment was used in the solvent groups. * *P* < 0.05, ** *P* < 0.01

To further investigate whether TPM1 was implicated in the YO-EV-induced bone formation process in vivo, we conducted a study wherein 3-month-old mice were administered with YO^si−Control^-EVs, YO^si−Tpm1^-EVs, or an equivalent quantity of solvent through intravenous injection once a week for one month, as similar with the frame in Fig. 3A. Consistent with our expectations, CT analysis demonstrated that Tpm1 inhibition considerably hampered the ability of YO-EVs to promote trabecular bone formation, characterized by considerably lower levels of Tb. BV/TV, Tb. N, and Tb. Th, as well as significantly higher levels of Tb. Sp in the YO^si−Tpm1^-EVs group in comparison to the YO^si−Control^-EVs group (Fig. 6A-B). However, there were no statistically significant variations in Ct. Th among mice treated with YO^si−Control^-EVs, YO^si−Tpm1^-EVs, or solvent (Fig. 6C). Furthermore, the three-point bending test showed that the femur ultimate load value tended to be lower in the YO^si−Tpm1^-EV group than in the YO^si−Control^-EVs group (Fig. 6D).

Immunohistochemical staining for OCN revealed that mice treated with YO^si−Control^-EVs exhibited a significant increase in osteoblast numbers. However, this favorable effect was nullified in mice treated with YO^si−Tpm1^-EVs (Fig. 6E-F). Additionally, blood OCN levels in mice treated with YO^si−Tpm1^-EVs were slightly lower than those in mice treated with YO^si−Control^-EVs (Fig. 6G). Immunofluorescence staining for PLIN demonstrated no significant changes in the number of marrow adipocytes in mice treated with YO^si−Control^-EVs. However, YO^si−Tpm1^-EVs slightly accelerated the formation of marrow adipocytes (Fig. 6H-I). The suggested explanation for these phenomena was that reduced F-actin polymerization enhanced adipogenesis in bone marrow microenvironments under the influence of various factors. These findings underscored the vital role of TPM1 in YO-EV-mediated bone formation.

## Discussion

**Fig. 7:**
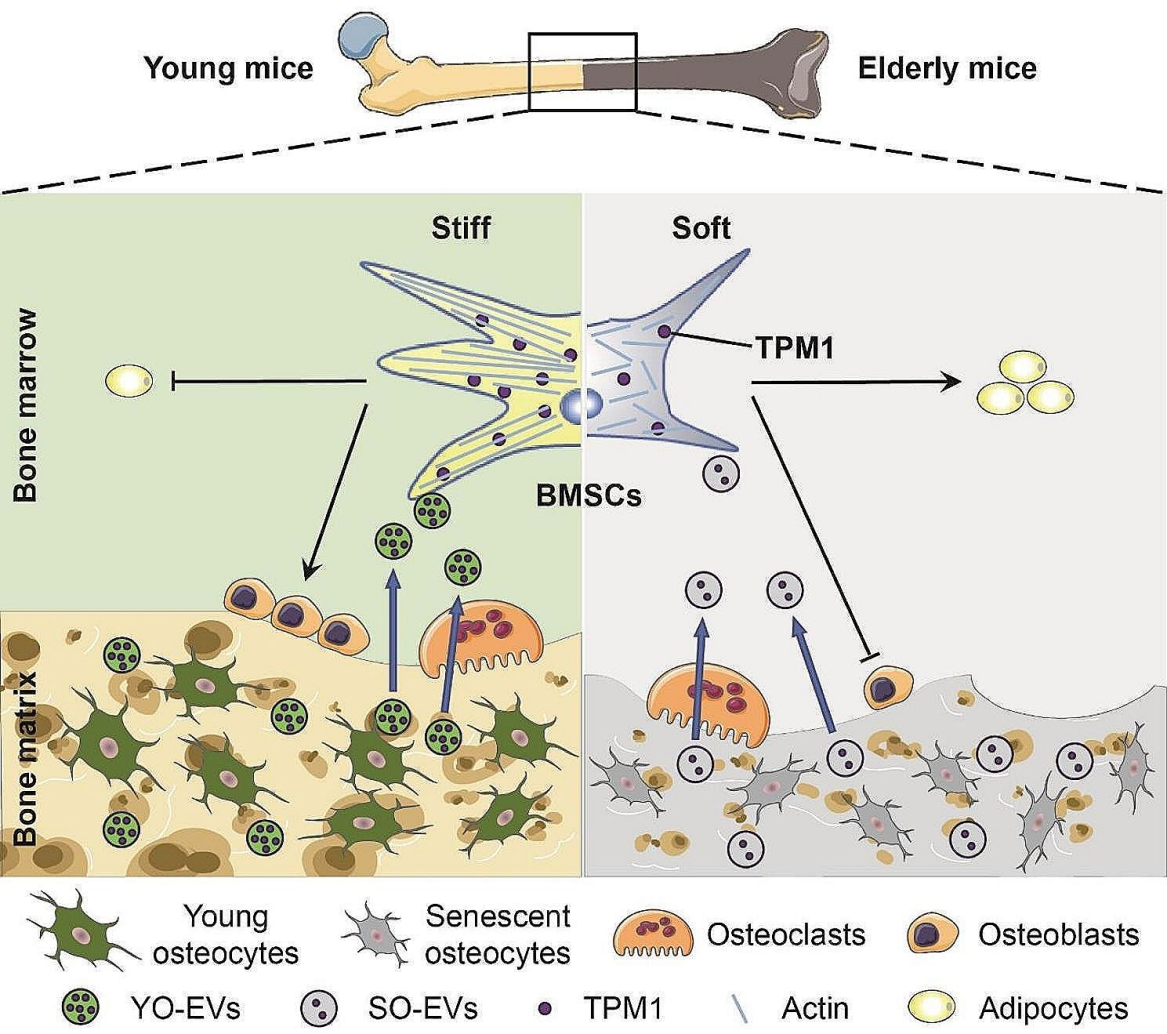
Schematic diagram illustrating the mechanism by which YO-EVs transfer TPM1 to facilitate BMSC osteogenesis. During bone resorption in young mice, YO-EVs are released from the bone matrix into the bone marrow, where they transfer TPM1 to promote osteogenic differentiation of BMSCs, rather than adipogenesis, by regulating F-actin polymerization. However, in elderly mice, a decrease in the delivery of TPM1 via SO-EVs leads to a bone-fat imbalance and, ultimately, osteoporosis. This schematic diagram provides a crucial visual representation of how osteocyte-derived EVs can affect bone homeostasis and underscores the importance of understanding the regulatory mechanisms involved in bone metabolism, particularly in regard to aging

Our study aimed to elucidate the role of osteocyte-derived EVs in bone remodeling, with a particular focus on understanding their impact on the differentiation of BMSCs. Our findings provide pivotal insights into the physiological mechanisms that underlie the increased incidence of osteoporosis with aging, emphasizing the crucial role of YO-EVs in maintaining bone microenvironment homeostasis. Notably, we discovered that YO-EVs transfer TPM1, a key regulator of F-actin polymerization, to promote osteogenic differentiation of BMSCs. This process appears to be compromised in older individuals, as evidenced in Fig. 7, suggesting a potential mechanism underlying age-related bone density loss.

Bone health undergoes dynamic changes over a lifetime, with peak bone mass usually reached in adolescence. Subsequently, bone mineral density (BMD) decreases, particularly in perimenopausal women and the elderly, leading to heightened risks of osteoporosis and fractures [29]. Osteoporosis, characterized by reduced bone mass and deteriorating bone microstructure, significantly increases bone fragility and fracture susceptibility [30]. Age-related changes in the bone microenvironment can negatively impact the behavior of BMSCs, ultimately affecting bone formation and remodeling [31, 32]. Our study sheds light on the complex interplay within the bone microenvironment, particularly highlighting the role of osteocyte-derived EVs and TPM1 in bone remodeling processes.

Extending beyond the traditional focus on osteoblasts and osteoclasts in anti-osteoporosis therapies [33, 34], our research spotlighted osteocytes, the most prevalent cells in bone tissue. Osteocytes, deeply embedded within the bone matrix, play a dynamic role in mineral homeostasis [17] and act as orchestrators of bone remodeling by modulating the activities of osteoblasts and osteoclasts [35, 36]. They also engage in paracrine signaling with BMSCs, influencing their differentiation and overall bone health [37]. Furthermore, recent studies have revealed that the transfer of matrix stiffening signals from osteocytes to surrounding cells plays a crucial role in regulating bone remodeling [38, 39].

Regarding this aspect, osteocyte-derived EVs have emerged as potential key players in modulating bone tissue stiffness and facilitating osteogenesis by carrying bioactive molecules that augment cell stiffness. Previous studies have indicated that osteocyte-derived EVs enhance osteoblastic activity by virtue of their constituents and have linked Ca2^+^-dependent contractions triggered by mechanical stimulation of osteocytes to the production and release of EVs containing bone regulatory proteins [25]. Our investigation revealed that YO-EVs significantly promote BMSC osteogenic differentiation, whereas SO-EVs stimulate BMSC adipogenic differentiation.

Bone remodeling is a vital process for maintaining bone health and involves the regulated activity of bone-forming osteoblasts and bone-resorbing osteoclasts [40]. In our study utilizing osteocyte-derived EV tracer mice, we observed abundant osteocyte-derived EVs in the bone matrix. One novel aspect of our study is the examination of the role of EVs released from the bone matrix during osteoclastic resorption. We observed that osteoclasts, when cultured with bone slices, enhanced the generation of EVs in the conditioned media. This finding is pivotal in understanding the mechanisms of bone remodeling and the release of factors that influence bone formation.

Our study highlights the crucial role of YO-EVs in regulating matrix stiffness and promoting osteogenic differentiation. Specifically, we observed that YO-EVs transfer the protein TPM1, a cytoskeletal protein [41–43], to BMSCs leading to increased matrix stiffness and upregulation of osteogenic markers in vitro. Furthermore, we found that TPM1 promotes cytoskeletal organization in BMSCs, increasing cell elongation and facilitating osteogenic differentiation. Remarkably, we also observed that SO-EVs exhibit reduced levels of TPM1 compared to YO-EVs, raising the possibility that EV-mediated signaling from osteocytes may contribute to age-related bone disorders. Our findings suggest that the modulation of TPM1 levels in bone microenvironment could be a potential therapeutic target for bone-related diseases [44, 45].

However, our study still has several limitations. A key area for future research is the long-term fate of internalized EVs in BMSCs. The long-term fate of internalized EVs is a less explored and challenging area in EV research. Given the complexity and variability of this process, further research is needed to elucidate the extended interactions and implications of EVs in bone metabolism and remodeling. Additionally, the conditioned medium contains a complex composition of paracrine biomolecules, EVs are just one among the complex array of paracrine mediators. For instance, osteoblast-lineage cells are known to modulate osteoclastic differentiation, however, both YO-EVs and SO-EVs had negligible effects on osteoclast differentiation. In the future, considering the broader array of paracrine mediators secreted by osteocytes could provide a more comprehensive understanding of bone remodeling and the development of age-related bone disorders.

## Conclusion

In conclusion, the findings of the present study have significant implications for the field of bone biology and the development of targeted therapeutic interventions intended to address various bone disorders, such as age-related osteoporosis. Specifically, the study highlights YO-EVs and TPM1 as critical modulators of bone formation and remodeling. By deepening our comprehension of the mechanisms that govern bone remodeling, we can explore potential new interventions that promote skeletal health and prevent bone-related ailments. The study’s outcomes underscore the significance of YO-EVs in regulating matrix stiffness and osteogenic differentiation, providing valuable insights into the regulatory mechanisms underlying bone homeostasis.

### Electronic supplementary material

Below is the link to the electronic supplementary material.

Supplementary Material 1

## Data Availability

The data supporting the findings of this study are available from the corresponding author upon reasonable request.

## Abbreviations

ALP: Alkaline phosphatase
ARS: Alizarin Red S
BMMs: Bone marrow macrophages/monocytes
BMSCs: Bone marrow mesenchymal stem cells
Col1a1: Collagen type I alpha 1 chain
Ct. Th: Cortical bone thickness
Ctsk: Cathepsin K
CTX-I: Cross-linked C-telopeptide of type I collagen
Dmp1: Dentin matrix acidic phosphoprotein 1
ELISA: Enzyme-linked immunosorbent assay
EVs: Extracellular vesicles
F-actin: Fibrous actin
µCT: Microcomputed tomography
NTA: Nanoparticle tracking analysis
OCN: Osteocalcin
ORO: Oil Red O
PLIN: Perilipin
Pparg: Peroxisome proliferator-activated receptor gamma
qRT−PCR: Quantitative real-time PCR
RANKL: Receptor activator for nuclear factor κB ligand
Runx2: Runt-related transcription factor 2
SEM: Scanning electron microscopy
siRNA: Small interfering RNA
SO-EVs: Senescent osteocyte-derived EVs
Tb. BV/TV: Trabecular bone volume fraction
Tb. N: Trabecular number
Tb. Sp: Trabecular separation
Tb. Th: Trabecular bone thickness
TEM: Transmission electron microscope
TPM1: Tropomyosin-1
TRAP: Tartrate-resistant acid phosphatase
YO-EVs: Young osteocyte-derived extracellular vesicles
