# Supplementary material for: Young osteocyte-derived extracellular vesicles facilitate osteogenesis by transferring tropomyosin-1
Source: J Nanobiotechnology. 2024 Apr 25;22:208. doi: 10.1186/s12951-024-02367-x (PMC11046877; doi:10.1186/s12951-024-02367-x)
Supplement: Supplementary file 1 — Supplementary Material 1 [file 12951_2024_2367_MOESM1_ESM.docx]

**Supplementary information**

**Young Osteocyte-derived Extracellular Vesicles Facilitate Osteogenesis by Transferring Tropomyosin-1**

Zhen-Xing Wang^1-3#^, Xiao Lin^4#^, Jia Cao^1-3^, Yi-Wei Liu^1,2^, Zhong-Wei Luo^1,2^, Shan-Shan Rao^1-3^, Qiang Wang^5^, Yi-Yi Wang^1,2^, Chun-Yuan Chen^1-3^, Guo-Qiang Zhu^1,2^, Fu-Xing-Zi Li^4^, Yi-Juan Tan^1,2^, Yin Hu^1^, Hao Yin^1,2^, You-You Li^6^, Ze-Hui He^1,2^, Zheng-Zhao Liu^1-3^, Ling-Qing Yuan^4^, Yong Zhou^6^, Zheng-Guang Wang^6^*, Hui Xie^1-3^*

* Correspondence authors: huixie@csu.edu.cn (H. Xie), wzg19830216@163.com (Z.G. Wang).

**Include:**

**Fig. S1. Characterization of primary osteocytes for osteocytic nature and senescent status.**

**Fig. S2. Internalization of YO-EVs and SO-EVs by BMSCs.**

**Fig. S3. Identification of EV tracer transgene *Dmp1^Cre^;Cd63^loxp-mCherry-loxp-eGFP^* mice.**

**Fig. S4. SEM images demonstrating osteoclast-induced resorption lacunae on bone slices.**

**Fig. S5. Distribution of YO-EVs and SO-EVs in bone tissue.**

**Fig. S6. The enriched molecular function (MF) terms of differentially expressed proteins between YO-EVs and SO-EVs.**

**Fig. S7. The enriched biological process (BP) terms of differentially expressed proteins between YO-EVs and SO-EVs.**

**Fig. S8. The enriched cell component (CC) terms of differentially expressed proteins between YO-EVs and SO-EVs.**

**Fig. S9. Impact of TPM1 Overexpression on Osteoblast and Adipogenic Differentiation of BMSCs.**

**
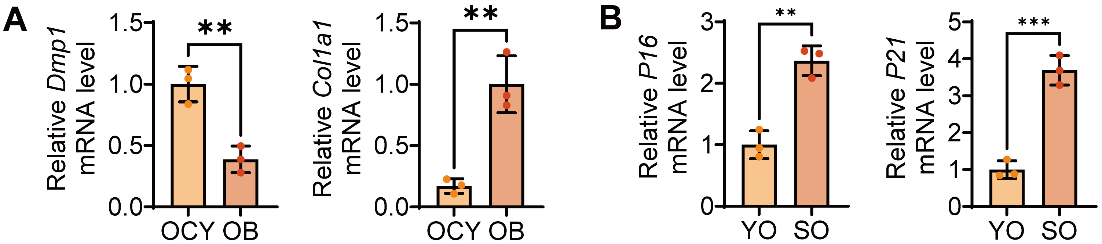
**

**Fig. S1. Characterization of primary osteocytes for osteocytic nature and senescent status.** (A) Gene expression analysis for *Dmp1* and *Col1a1* in osteocytes (OCY) and osteoblasts (OB). (B) Gene expression analysis for *P16* and *P21* in young osteocytes (YO) and senescent osteocytes (SO). n = 3 per group. ** *P* < 0.01, *** *P* < 0.001.

**
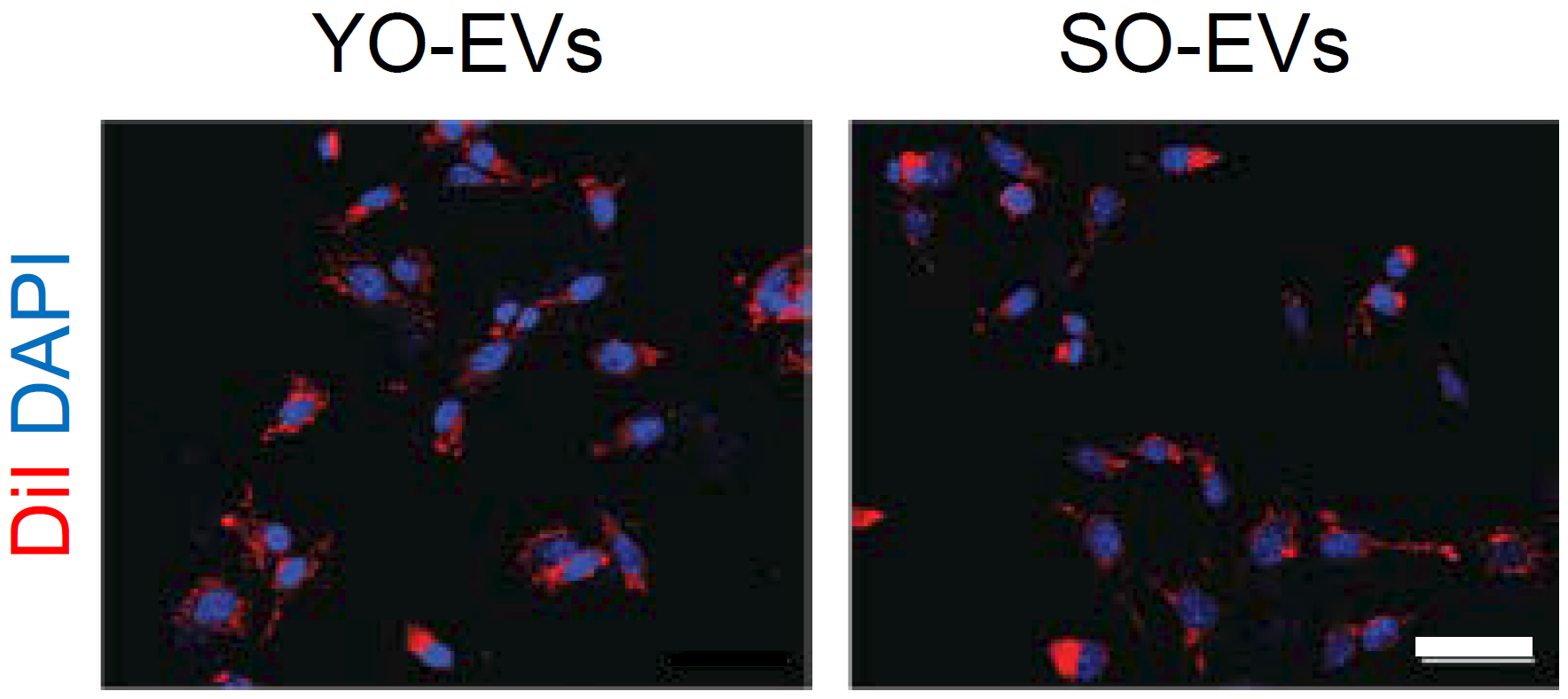
**

**Fig. S2. Internalization of YO-EVs and SO-EVs by BMSCs.** Representative fluorescent microscopic images of red florescent dye DiI-labeled YO-EVs and SO-EVs internalization by BMSCs. The cell nucleus was stained with DAPI (blue). Scale bar: 20 µm.


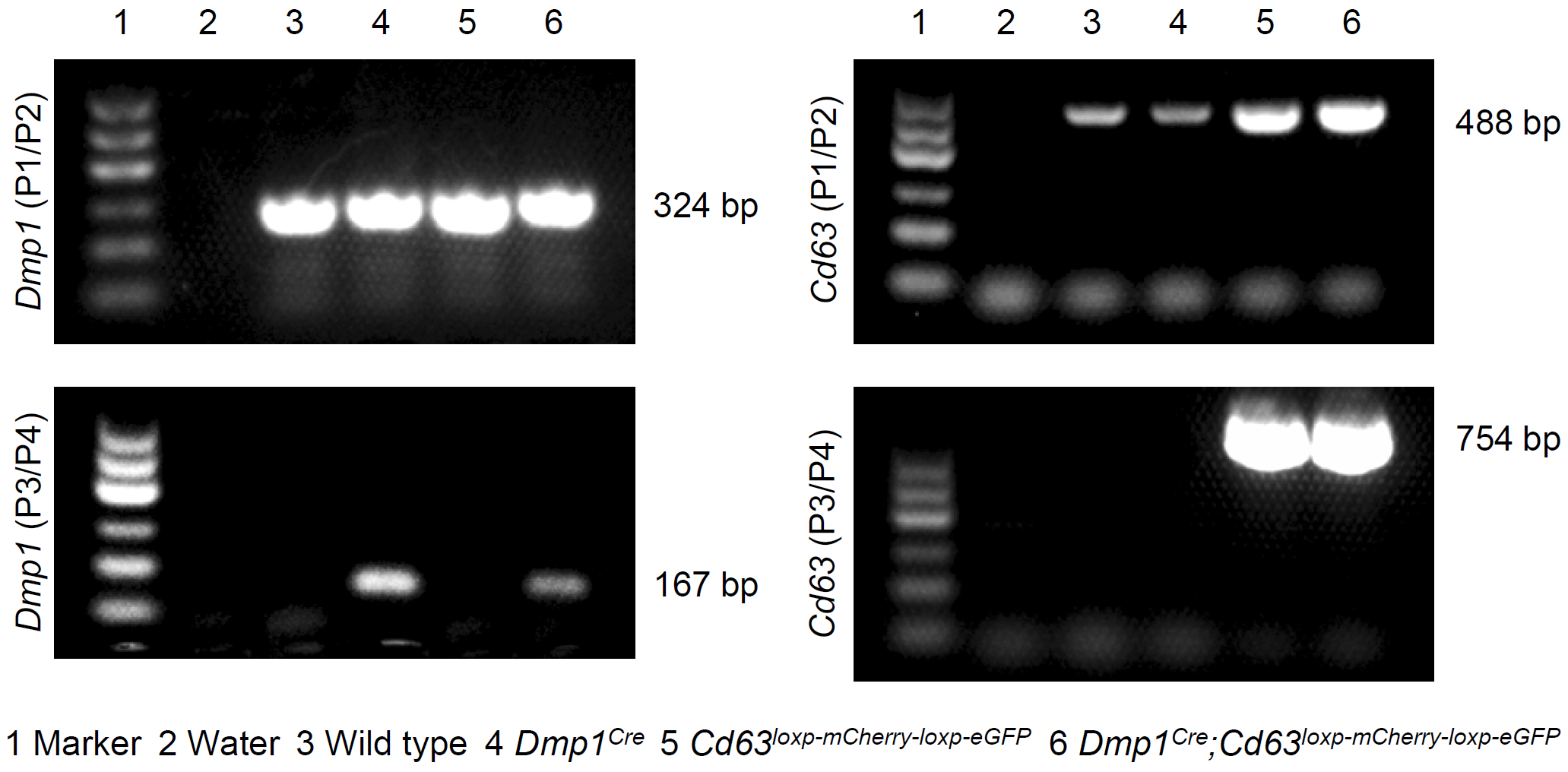


**Fig. S3. Identification of EV tracer transgene *Dmp1^Cre^;Cd63^loxp-mCherry-loxp-eGFP^* mice.** PCR genotyping of wild type mice, *Dmp1^Cre^* mice, *Cd63^loxp-mCherry-loxp-eGFP^* mice, and *Dmp1^Cre^;Cd63^loxp-mCherry-loxp-eGFP^* mice using primers for determining the insertion of Cre (left) and eGFP (right).


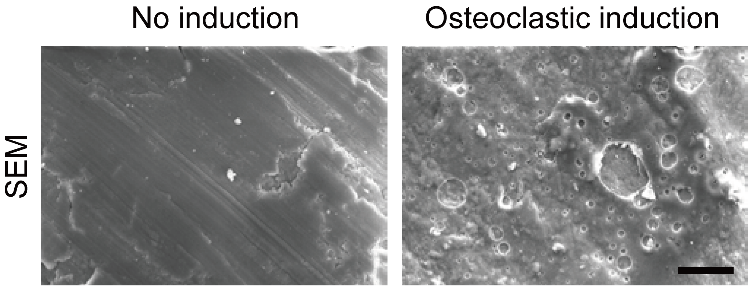


**Fig. S4. SEM images demonstrating osteoclast-induced resorption lacunae on bone slices.** SEM images presents the surface morphology of bone slices following exposure to osteoclast cultures for 14 days. Scale bar: 20 µm.


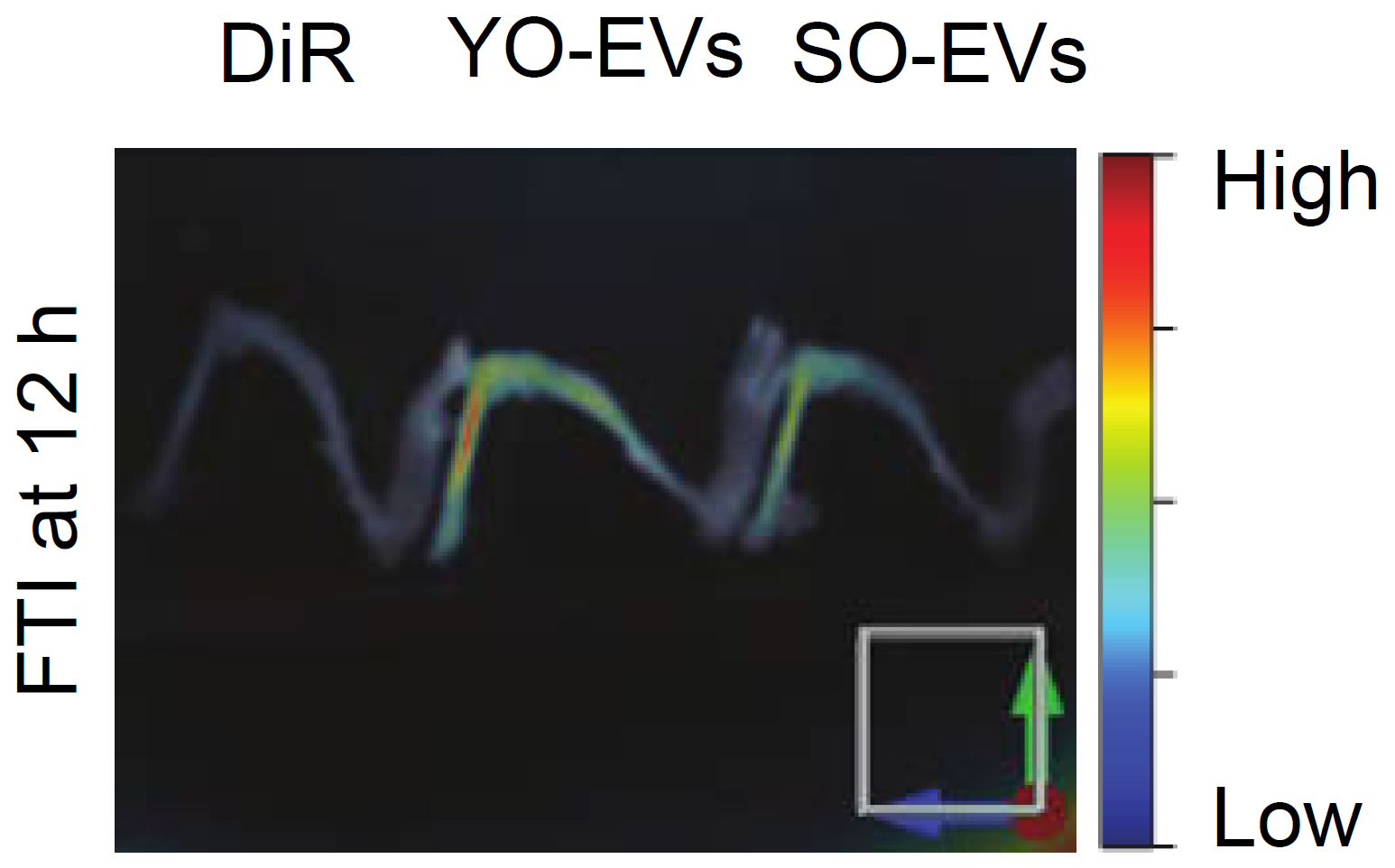


**Fig. S5. Distribution of YO-EVs and SO-EVs in bone tissue.** Representative *ex vivo* fluorescent images of lower limb bone from wild type mice treated with DiR-labeled YO-EVs and SO-EVs or solvent by intravenous administration.


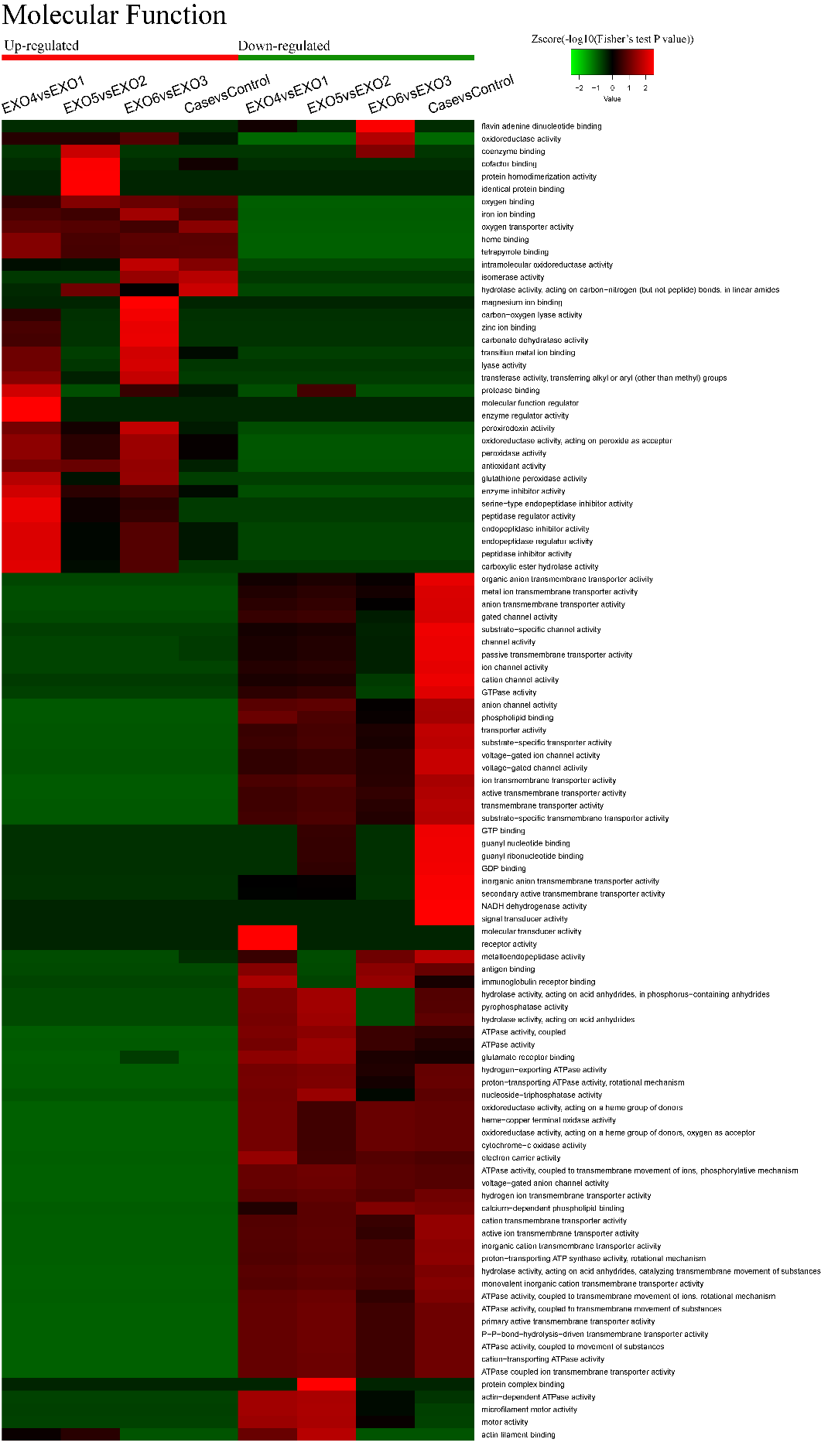


**Fig. S6. The enriched molecular function (MF) terms of differentially expressed proteins between YO-EVs and SO-EVs.** The difference is greater than 1.5-fold, *P* < 0.05.


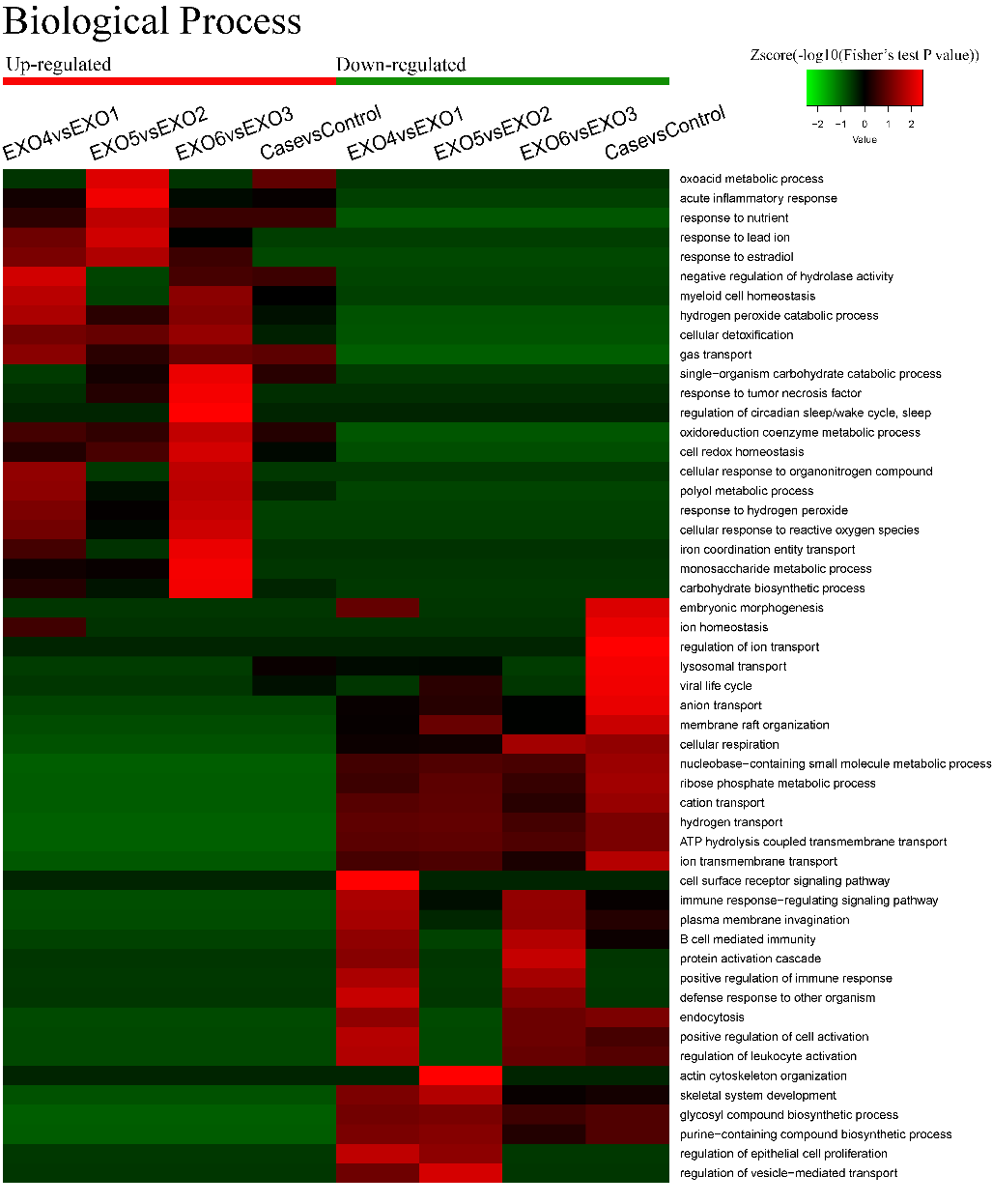


**Fig. S7. The enriched biological process (BP) terms of differentially expressed proteins between YO-EVs and SO-EVs.** The difference is greater than 1.5-fold, *P* < 0.05.


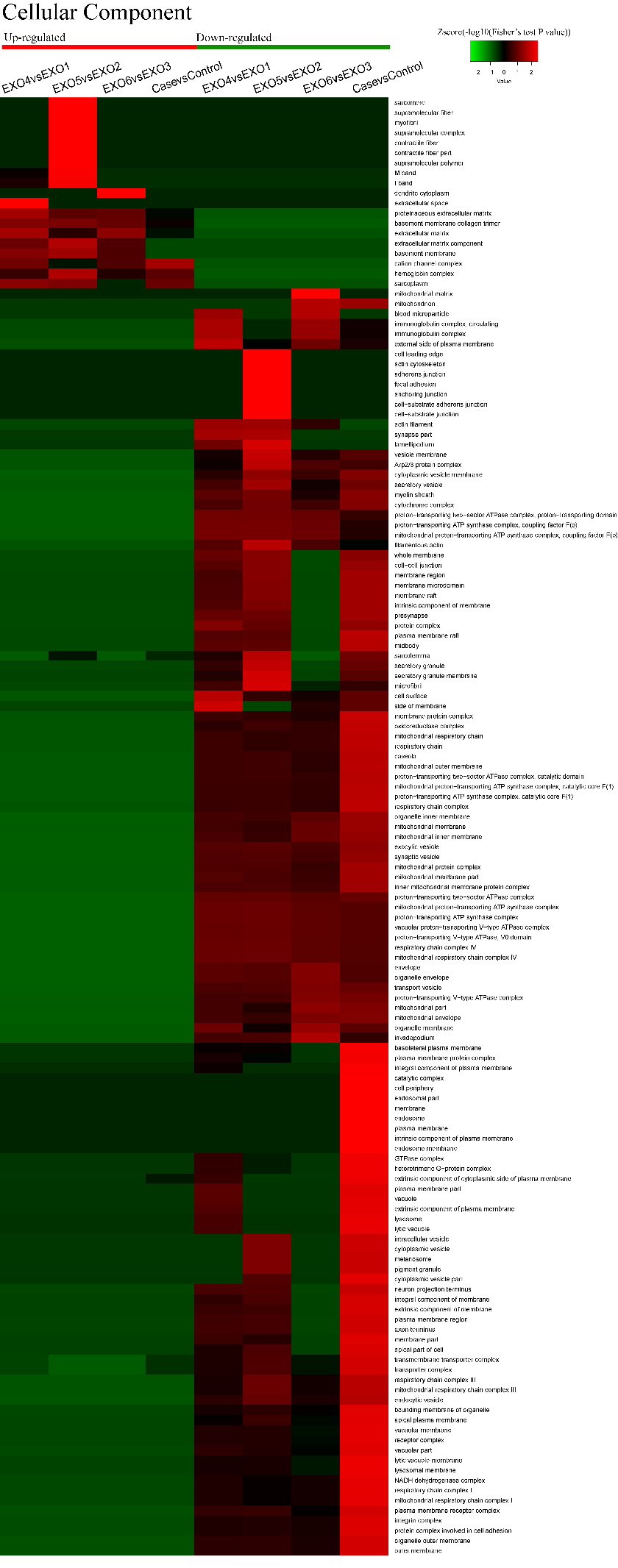


**Fig. S8. The enriched cell component (CC) terms of differentially expressed proteins between YO-EVs and SO-EVs.** The difference is greater than 1.5-fold, *P* < 0.05.


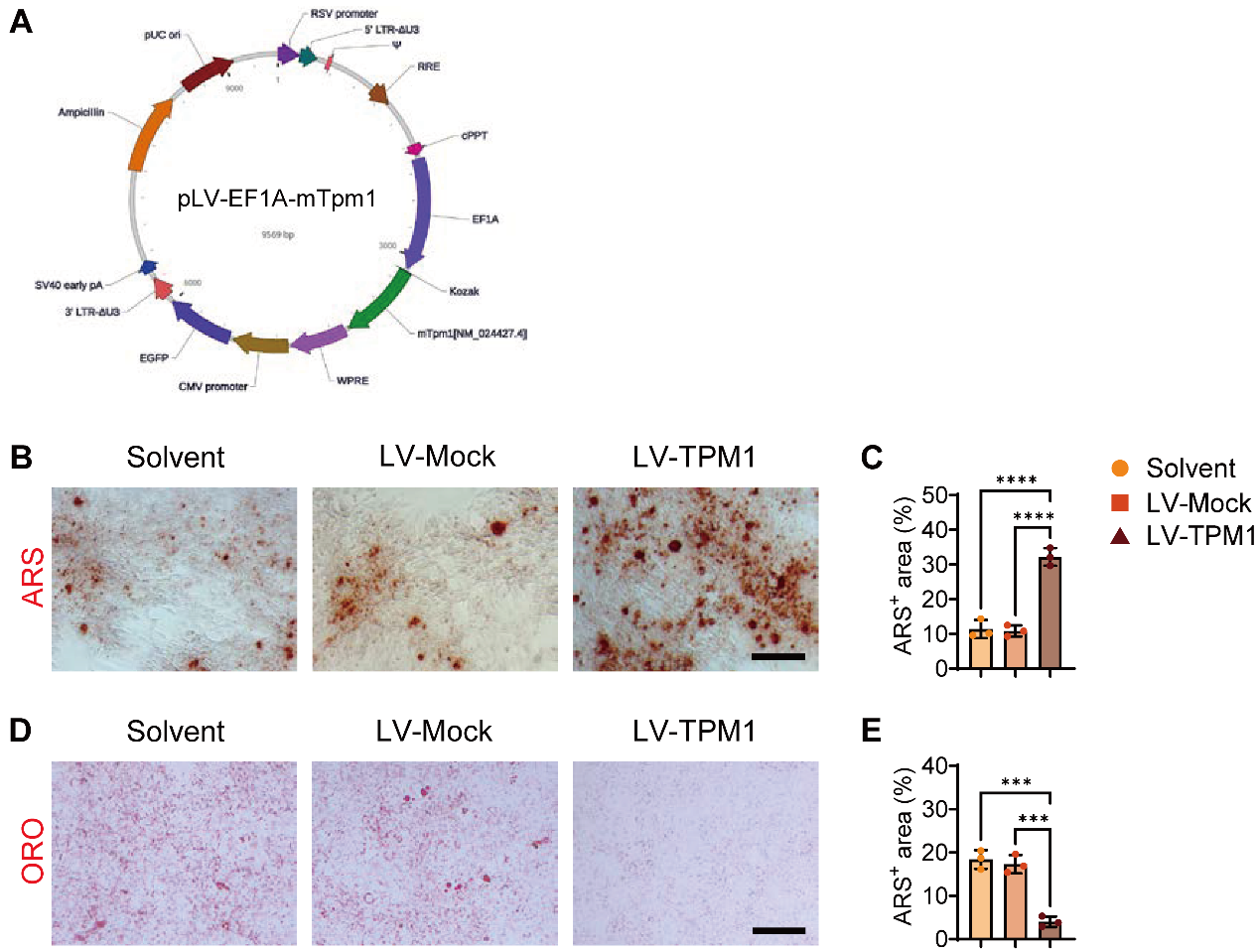


**Fig. S9. Impact of TPM1 Overexpression on Osteoblast and Adipogenic Differentiation of BMSCs.** (A) Illustration of the constructed lentiviral vector used for overexpressing Tropomyosin 1 (TPM1). (B-C) ARS staining (B; red) and quantification of the percentages of ARS^+^ area (C) of BMSCs treated with Solvent, LV-Mock, or LV-TPM1 under osteogenic induction. (D-E) ORO staining (D; red) and quantification of the percentages of ORO^+^ area (E) of BMSCs treated with Solvent, LV-Mock, or LV-TPM1 under adipogenic induction. Scale bar: 50 μm. n = 3 per group. PBS treatment was used in the solvent groups. *** *P* < 0.001, **** *P* < 0.0001.
